# A meta-analysis of the watch-and-wait strategy versus total mesorectal excision for rectal cancer exhibiting complete clinical response after neoadjuvant chemoradiotherapy

**DOI:** 10.1186/s12957-021-02415-y

**Published:** 2021-10-18

**Authors:** Guilin Yu, Wenqing Lu, Zhouguang Jiao, Jun Qiao, Shiyang Ma, Xin Liu

**Affiliations:** 1 Department of General Surgery, Cancer Hospital of China Medical University, Liaoning Cancer Hospital and Institute, No. 44 Xiaoheyan Road, Dadong District, Shenyang, 110042 Liaoning Province People’s Republic of China; 2 School of Life Sciences, Hebei University, Baoding, 071002 Hebei Province People’s Republic of China; 3 Institute of Process Engineering, Chinese Academy of Science, Beijing, 100190 People’s Republic of China; 4 Department of Colorectal Surgery, Cancer Hospital of China Medical University, Liaoning Cancer Hospital and Institute, No. 44 Xiaoheyan Road, Dadong District, Shenyang, 110042 Liaoning Province People’s Republic of China

**Keywords:** Watch-and-wait, Complete clinical response, Total mesorectal excision, Rectal cancer, Meta-analysis

## Abstract

**Background:**

Some clinical researchers have reported that patients with cCR (clinical complete response) status after neoadjuvant chemoradiotherapy (nCRT) could adopt the watch-and-wait (W&W) strategy. Compared with total mesorectal excision (TME) surgery, the W&W strategy could achieve a similar overall survival. Could the W&W strategy replace TME surgery as the main treatment option for the cCR patients? By using the meta-analysis method, we evaluated the safety and efficacy of the W&W strategy and TME surgery for rectal cancer exhibiting cCR after nCRT.

**Methods:**

We evaluated two treatment strategies for rectal cancer with cCR after nCRT up to July 2021 by searching the Cochrane Library, PubMed, Wanfang, and China National Knowledge Infrastructure (CNKI) databases. Clinical data for primary outcomes (local recurrence, cancer-related death and distant metastasis), and secondary outcomes (disease-free survival (DFS) and overall survival (OS)) were collected to evaluate the efficacy and safety in the two groups.

**Results:**

We included nine studies with 818 patients in the meta-analysis, and there were five moderate-quality studies and four high-quality studies. A total of 339 patients were in the W&W group and 479 patients were in the TME group. The local recurrence rate in the W&W group was greater than that in the TME group in the fixed-effects model (OR 8.54, 95% CI 3.52 to 20.71, *P* < 0.001). The results of other outcomes were similar in the two groups.

**Conclusion:**

The local recurrence rate of the W&W group was greater than that in the TME group, but other results were similar in the two groups. With the help of physical examination and salvage therapy, the W&W strategy could achieve similar treatment effects with the TME approach.

**Trial registration:**

Protocol registration number: CRD42021244032.

**Supplementary Information:**

The online version contains supplementary material available at 10.1186/s12957-021-02415-y.

## Background

Given its high incidence and fatality rate, rectal cancer seriously endangers human health [1]. To ensure the radical resection of the tumor, some patients with low rectal cancer need to have their anus removed [2]. The major trauma creates physical and psychological problems for the patients [3]. Neoadjuvant chemoradiotherapy (nCRT) combined with surgery has become the standard treatment mode for the locally advanced rectal cancer. nCRT can reduce the local recurrence and tumor size in patients with locally advanced rectal cancer [4]. In addition, it can increase the chance of preserving the anus by shrinking and downgrading tumors [5]. Multiple colorectal cancer guidelines recommend TME after nCRT as the standard treatment for locally advanced rectal cancer, but TME surgery has various complications, such as bleeding, intestinal obstruction, anastomotic leakage, and other complications [6]. TME could also have a long-term negative impact on the defecation control and sexual functions [7, 8].

Approximately 20% rectal cancer patients could have complete tumor regression after nCRT, and the specific phenomenon is defined as clinical complete response (cCR) [9]. In 2004, the study of Habr-Gama et al. proposed that patients with cCR status could adopt the watch-and-wait (W&W) treatment strategy. Since then, a series of clinical researches have promoted the discussion about the treatment strategy of cCR patients [10, 11]. Compared with TME surgery, the W&W strategy could achieve a similar overall survival and a better preservation of organ anatomy and physiological function [12]. Some related meta-analyses had published. The results indicated that the W&W group exhibited a higher local recurrence rate than the TME group, but the overall survival was similar in the two groups [13, 14]. In our study, we included a larger number of studies with no difference in the baseline data (the available of clinical data pre-T stage, pre-N stage, pre-TNM stage, and preclinical stage had no significant difference, *P >* 0.05) to assess the advantages and disadvantages between the two approaches. We compared the primary outcomes (local recurrence, cancer-related death, and distant metastasis) and secondary outcomes (DFS and OS) to evaluate the efficacy and safety in the two approaches. The results of our study provide clinical evidence for the treatment of locally advanced rectal cancer.

## Methods

### Literature search

Our research method has completed the protocol registration in PROSPERO (Protocol registration number: CRD42021244032; the link to the protocol: https://www.crd.york.ac.uk/prospero/#recordDetails). The more details were in the supplementary material 1 and 2. We performed the meta-analysis according to the PRISMA guidelines (The more details were in the supplementary material 3). The specific information of the PICOS included population, intervention, comparator and outcomes. Population: rectal cancer patients achieved cCR response after nCRT; intervention: watch-and-wait strategy; comparator: total mesorectal excision; outcomes: primary outcomes (local recurrence, cancer-related death, and distant metastasis) and secondary outcomes (DFS and OS) (The more details were in the supplementary material 4).

We performed a systematic search of the PubMed, Cochrane Library, Embase, CNKI (China National Knowledge Infrastructure), and Wanfang databases to obtain relevant literature (up to July 2021). The search string was built as follows: “watch-and-wait” or “non-operative management” or “total mesorectal excision” or “neoadjuvant chemoradiotherapy” and “rectal cancer”. Information on the search terms is shown in supplementary material 5.

### Inclusion and exclusion criteria

The study had four inclusion criteria: the study had consistent baseline database and the available clinical data between the two groups (the available clinical data pre-T stage, pre-N stage, pre-TNM stage and pre-clinical stage had no significant difference, *P* > 0.05); total mesorectal excision included abdominal-perineal resection (APR), Dixon and other radical surgical approaches, but not local excision; rectal cancer patients (stage I to III) with cCR status after nCRT and randomized Controlled Trial (RCTs), retrospective comparative non-randomized studies (RCNTs), prospective comparative non-randomized studies (PCNTs), cohort studies, or case-control studies. The details of neoadjuvant treatment and the diagnostic criteria of each method in each study are shown in supplementary material 6 and 7. The exclusion criteria were as follows: a significant difference in baseline data and no valuable information of the study; as well as rectal cancer patients who did not achieve cCR status after nCRT; and reviews, case reports, or other unsuitable types.

### Data extraction and quality control

The clinically useful data were collected by two reviewers independently according to the Newcastle-Ottawa Scale (NOS) guidelines [15]. Any disagreement was resolved by discussion until consensus was reached or consulted by a third author. XL, ZGJ, and SYM performed the literature search and collected the data. We conducted a preliminary sorting of the referenced documents to exclude duplicate documents. Then, we read the title and abstract of the article, and excluded studies with no control group, no rectal cancer, and incomplete data. Finally, the full text was read and the final documents were screened out. The details of the collected data are listed in the tables. Table 1 mainly contains basic information, such as: the first author, publication data, author's country, patient age, mean tumor diameter, mean distance from the anal verge, study size and study type. Table 2 contains clinical stage, T stage, N stage and the clinical staging after nCRT. Table 3 contains long-term outcomes of the patients. The primary and secondary outcomes are mainly shown in Table 3. LE, CRD and DM were the primary outcomes of the study, whereas 2-year, 3-year, and 5-year DFS and OS were the secondary outcomes. The other information of basic information are shown in supplementary material 8. The details of salvage therapy in the W&W group are shown in supplementary material 9. We tried to use various methods to obtain more missing data, but we failed to acquire more valuable data.

**Table 1:** Characteristics of the included articles

Study	Country	Year	Case		Age		Mean Tumor diameter (cm)		Mean distance from anal verge (cm)		Adjuvant chemotherapy (perform,n)	
			W&W	TME	W&W	TME	W&W	TME	W&W	TME	W&W	TME
Ayloor[16]	India	2013	23	10	50 (25-71)	55 (30-69)	NR	NR	3 (0-6)	4 (0-7)	NR	NR
Dalton[17]	UK	2012	6	6	64 (54-71)	69 (38-88)	5.5 (2.81)	6.1 (2.13)	4.6 (3.05)	5.5 (2.39)	NR	NR
Habr[18]	Brazil	2004	71	22	58.1 (35-92)	53.6 (25-73)	3.6 (1-7)	4.2 (2.5-7)	3.6 (0-7)	3.8 (2-7)	NR	NR
Lai[19]	Taiwan	2016	18	26	67.5 (15.20)	63.7 (14.05)	NR	NR	3.3	4.8	NR	NR
Li[20]	China	2015	30	92	62 (55-82)	56 (34-73)	NR	NR	3.5 (0-7)	3.8 (0-7)	NR	NR
Mass[21]	Netherlands	2011	21	20	65±9	66±10	NR	NR	2.8	3.3	16	14
Smith[22]	USA	2015	17	30	62.3	60.4	NR	NR	4.1	6	11	18
Wang[23]	China	2020	59	179	58 (29-78)	57 (27-83)	NR	NR	NR	NR	NR	NR
Wang[24]	China	2021	94	94	57.5 (46-65)	56 (49-62.3)	NR	NR	4 (3-5)	4 (3-5)	NR	NR
Study	Follow-up time (month)		Radical surgery type		Quality control			Study design				NOS score
	W&W	TME			selection	comparability	outcome	RNCT	PNCT		RCT	
Ayloor[16]	72	72	APR or LAR		3	2	1	√	-		-	6
Dalton[17]	25.3	39.3	TME		3	2	2	-	√		-	7
Habr[18]	57.3	48	TME		2	3	1	-	√		-	6
Lai[19]	49 (21.9)	42 (17.9)	APR or LAR or LAR+ loop stoma		2	2	2	√	-		-	6
Li[20]	58	58	APR or LAR		3	1	2	-	√		-	6
Mass[21]	25±19	35±23	TME		3	2	2	-	√		-	7
Smith[22]	68.4	66.3	APR or LAR		2	3	2	√	-		-	7
Wang[23]	60	60	TME		2	2	2	√	-		-	6
Wang[24]	38.2	38.2	TME		3	2	2	√	-		-	7

Notes: PNCT: prospective non-randomized controlled trial; RNCT: retrospective non-randomized controlled trial; 5-FU:5-Fluorouracil; Cape: Capecitabine; W&W: watch and wait; TME: total mesorectal excision; APR: abdominal-perineal resection; LAR: Low anterior resection. NR: no record

The orders of additional information were range, standard deviation, percentage or NR (if not reported)

**Table 2:** clinical stage, T stage and N stage and Pathlologic T stage of the included articles

Study	Clinical stage														
	I				II				III					IV	
	W&W		TME		W&W		TME		W&W		TME			W&W	TME
Ayloor[17]	NR		NR		NR		NR		NR		NR			NR	NR
Dalton[18]	NR		NR		NR		NR		NR		NR			NR	NR
Habr[19]	NR		NR		NR		NR		NR		NR			NR	NR
Lai[20]	NR		NR		11		8		7		18			NR	NR
Li[22]	NR		NR		NR		NR		NR		NR			NR	NR
Mass[23]	NR		NR		NR		NR		NR		NR			NR	NR
Smith[26]	1		2		10		16		6		12			0	0
Wang[28]	8		2		17		26		25		49			NR	NR
Wang[28]	NR		NR		NR		NR		NR		NR			NR	NR
Study	T stage				N stage				Pathlologic T stage					Pathlologic N stage	
	T1-T2		T3-T4		N0		N1-N2								
	W&W	TME	W&W	TME	W&W	TME	W&W	TME	ypT0	ypT1	ypT2	ypT3	ypT4	ypN0	ypN1-N2
Ayloor[16]	9	4	14	6	NR	NR	NR	NR	6	NR	3	1	NR	6	4
Dalton[17]	1	NR	5	6	1	NR	5	6	NR	NR	NR	NR	NR	NR	NR
Habr[18]	14	1	57	21	55	16	16	6	NR	NR	NR	NR	NR	NR	NR
Lai[19]	NR	NR	NR	NR	NR	NR	NR	NR	NR	NR	NR	NR	NR	NR	NR
Li[20]	8	24	22	68	14	39	16	53	NR	NR	NR	NR	NR	NR	NR
Mass[21]	6	1	15	19	6	3	15	17	NR	NR	NR	NR	NR	NR	NR
Smith[22]	2	4	15	26	11	18	6	12	NR	NR	NR	NR	NR	NR	NR
Wang[23]	6	8	53	171	14	47	45	132	NR	NR	NR	NR	NR	NR	NR
Wang[24]	9	8	85	86	19	24	75	70	NR	NR	NR	NR	NR	NR	NR

NR:no record

**Table 3:** Primary and secondary outcomes of the included articles

Study	LR (n/%)		DM (n/%)		CRD (n/%)		2-year OS (n/%)		2-year DFS (n/%)	
	W&W	TME	W&W	TME	W&W	TME	W&W	TME	W&W	TME
Ayloor[16]	7(30.13%)	0	3(13.04%)	2(20%)	NR	NR	NR	NR	NR	NR
Dalton[17]	NR	NR	NR	NR	NR	NR	6(100%)	6(100%)	6(100%)	6(100%)
Habr[18]	2(2.81%)	0	3(4.22%)	3(13.6%)	0	2(9.09%)	71(100%)	20(90.9%)	70(98.59%)	19(86.36%)
Lai[19]	2(11.11%)	0	0	1(3.84%)	NR	NR	18(100%)	26(100%)	NR	NR
Li[20]	2(6.66%)	2(2.17%)	1(3.33%)	5(5.43%)	0	4(4.34%)	30(100%)	92(100%)	29(96.66%)	91(98.91%)
Mass[21]	1(4.76%)	0	0	1(5%)	NR	NR	21(100%)	19(95%)	19(90.47%)	19(95%)
Smith[22]	1(5.55%)	0	1(5.55%)	1(3.33%)	NR	NR	17(100%)	30(100%)	16(94.11%)	29(96.66%)
Wang[23]	7(11.86%)	1(0.56%)	6(10.16%)	17(9.49%)	NR	NR	NR	NR	NR	NR
Wang[24]	14(14.89%)	1(1.06%)	20(21.27%)	11(11.7%)	4(4.2%)	6(6.3%)	NR	NR	NR	NR
Total	36(10.81%)	4(0.84%)	34(10.21%)	41(8.66%)	4(2.1%)	12(5.76%)	163(100%)	193(98.4%)	140(96.55%)	164(96.47%)
Study	3-year OS (n/%)		3-year DFS (n/%)			5-year OS (n/%)			5-year DFS (n/%)	
	W&W	TME	W&W	TME		W&W	TME		W&W	TME
Ayloor[16]	NR	NR	NR	NR		NR	NR		NR	NR
Dalton[17]	NR	NR	NR	NR		NR	NR		NR	NR
Habr[18]	NR	NR	NR	NR		71(100%)	20(90.90%)		68(95.77%)	19(86.36%)
Lai[19]	NR	NR	NR	NR		18(100%)	24(92.30%)		NR	NR
Li[20]	NR	NR	NR	NR		30(100%)	88(95.65%)		27(90%)	85(92.39%)
Mass[21]	21(100%)	19(95%)	20(95.2%)	19(95%)		NR	NR		NR	NR
Smith[22]	17(100%)	29(96.66%)	15(88.23%)	29(96.66%)		17(100%)	29(96.66%)		15(88.23%)	29(96.66%)
Wang[23]	59(100%)	175(97.9%)	NR	NR		53(89.83%)	175(97.76%)		NR	NR
Wang[24]	93(99%)	90(96%)	93(99%)	89(95%)		NR	NR		NR	NR
Total	190(99.47%)	313(96.9%)	128(96.96%)	137(95.14%)		189(96.92%)	336(96.28%)		110(93.22%)	133(92.36%)

Notes: LR: local recurrence; DM: distant metastasis; CRD:cancer related death; DFS: disease free survival; OS: overall survival; NR:no record

### Quality assessment

We assessed the qualities of the included studies by using the NOS assessment scale (Newcastle-Ottawa Quality Assessment Scale). Study qualities were classified as high level (7 ≤ scores ≤ 9), middle level (4 < scores ≤ 6), and low level (1 < scores ≤ 3). We included five moderate-quality studies and four high-quality studies, and the nine included studies were five RCNT and four PNCT. We did not find any RCTs through a literature search. The details are shown in Table 1.

### Statistical analysis

We performed the meta-analysis by using RevMan 5.0 and Stata 11.0 software. Continuous data and dichotomous data were evaluated by the standardized mean differences (SMDs) and relative risks (ORs or RRs) with 95% confidence intervals respectively. We used the *I*^2^ statistic and funnel plots to assess the heterogeneity and publication bias separately. We used random effects models to analyze the data with huge heterogeneity (*I*^2^ ≧ 50%) and the fixed-effects model for little heterogeneity (*I*^2^ < 50%).

## Results

### Study selection

**Fig. 1:**
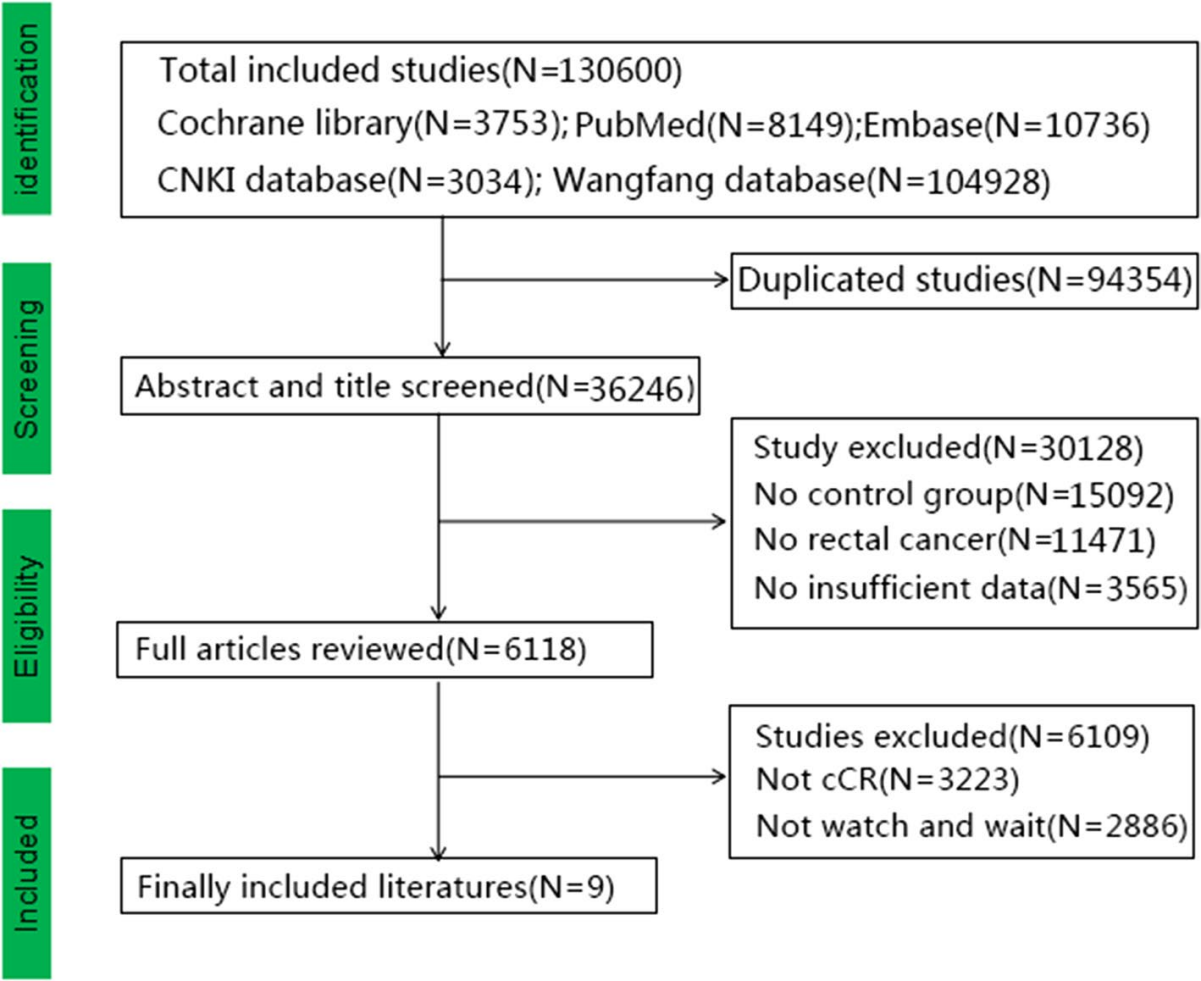
Flowchart of the included studies

After we completed the relevant search, we deleted duplicate studies (*N* = 94354), as well as studies with insufficient data (*N* = 3565), no control group (*N* = 15092), no rectal cancer (*N* = 11471), not-cCR (*N* = 3223), and not-W&W (*N* = 2886) (Fig. 1). After the literature screening, a total of nine studies with 818 patients were included [16–24]. A total of 339 patients were in the W&W group, and 479 patients were in the TME group. The meta-analysis included five Eastern studies and four Western studies. Eight suitable English studies and one suitable Chinese study (Wang^23^) were identified. Statistical methods used in the included studies were *χ*^2^ and *t* tests. Ayloor et al. [16] and Dalton et al. [17] did not describe statistical methods in the article. Baseline characteristics of other studies have been statistically analyzed, and there was no significant differences in the baseline characteristics (such as pretreatment T stage, N stage, TNM stage) [25–29]. The study of Smith et al. [22] included one stage IV patient. We excluded the stage IV patients and screened valuable data for further research. The details of the included studies are shown in Table 1 and supplementary material 8. A total of 86.4% of patients with T3–4 stage disease and 63.9% of patients with N1-2 stage disease were included before nCRT (Table 2). Only the study of Ayloors et al. [16] reported the clinical stage after nCRT, and most tumors were clinically downgraded. We hypothesized that the majority of the patients had tumor downstaging after nCRT (Table 2). The details of long-term outcomes (local recurrence, distant metastasis, cancer-related death, disease-free survival, and overall survival) are shown in Table 3. The details of salvage therapy and more information on the W&W group are shown in supplementary material 10 and 11. A total of 36 patients had local recurrence in the W&W group and 30 (83.33%) underwent salvage therapy. TME surgery was the main type of salvage therapy for local recurrence. Twelve (40%) patients underwent APR surgery, and the overall survival times in the Ayloor and Li studies were 66 and 49.5 months, respectively.

### W&W group versus the TME group

#### Primary outcomes

##### Local recurrence, distant metastasis, and cancer-related death

**Fig. 2:**
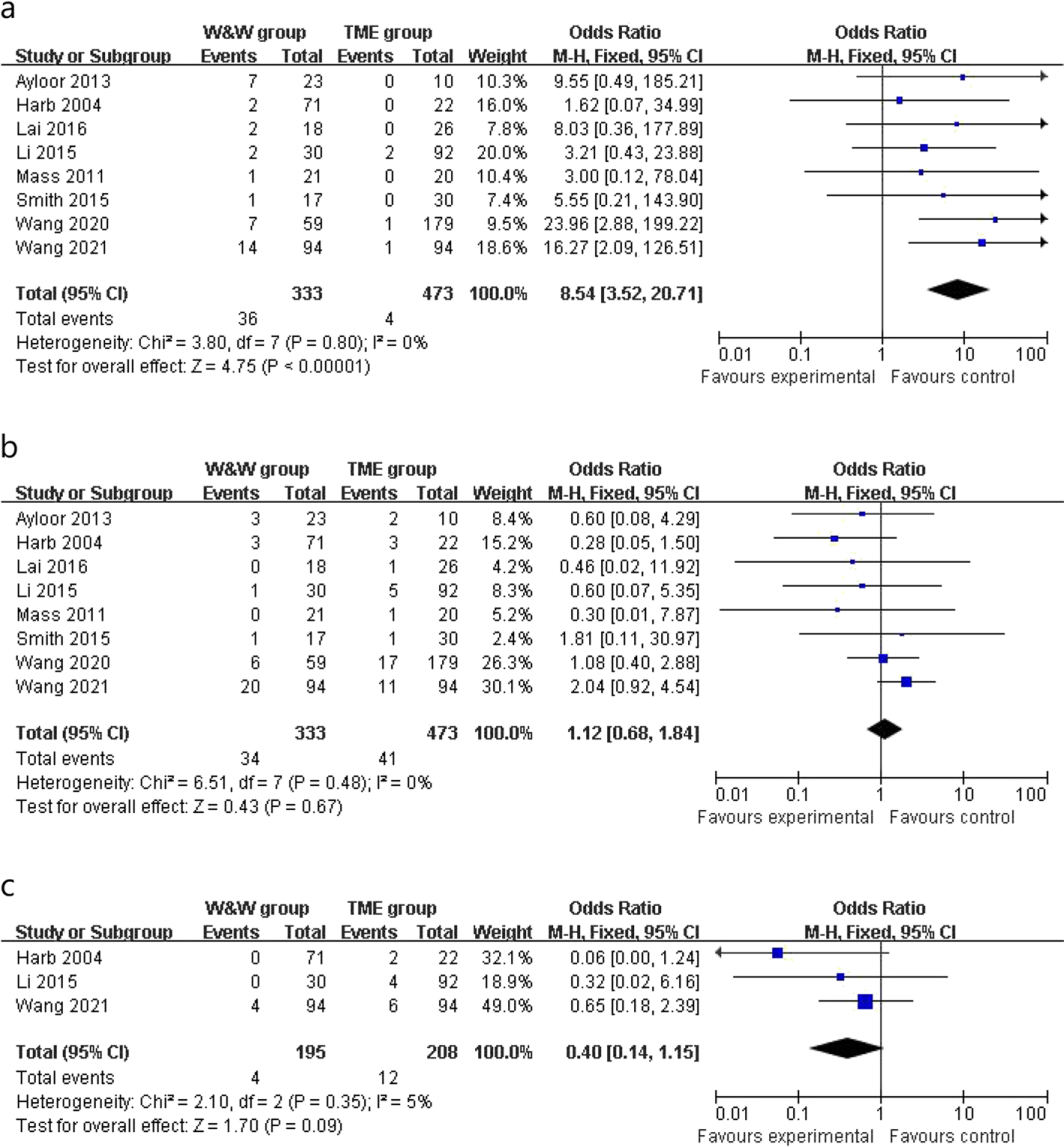
Outcomes of W&W group versus TME group. **a** Local recurrence. **b** Distant metastasis. **c** Cancer-related death

Clinical data of the local recurrence rate were reported in 8 studies, and the local recurrence rate of the W&W group was greater than that of the TME group in the fixed-effects model (OR 8.54, 95% CI 3.52 to 20.71, *P* < 0.001, *χ*^2^ = 3.80, *P* = 0.80, *I*^2^ = 0%, 10.81% vs 0.84%, Fig. 2a). The distant metastasis rate was similar in the W&W group and the TME group in the fixed-effects model with little heterogeneity (OR 1.12, 95% CI 0.68 to 1.84, *P* = 0.67, *χ*^2^ = 6.51, *P* = 0.48, *I*^2^ = 0%, 10.21% vs 8.66%, Fig. 2b). Cancer-related death (OR 0.40, 95% CI 0.14 to 1.15, *P* = 0.35, *χ*^2^ = 2.10, *P* = 0.35, *I*^2^ = 5%, 2.05% vs 5.76%, Fig. 2c) was similar between the two groups in the fixed-effects model with high heterogeneity.

#### Secondary outcomes 2-year OS, 2-year DFS, 3-year OS, 3-year DFS, 5-year OS, and 5-year DFS

**Fig. 3:**
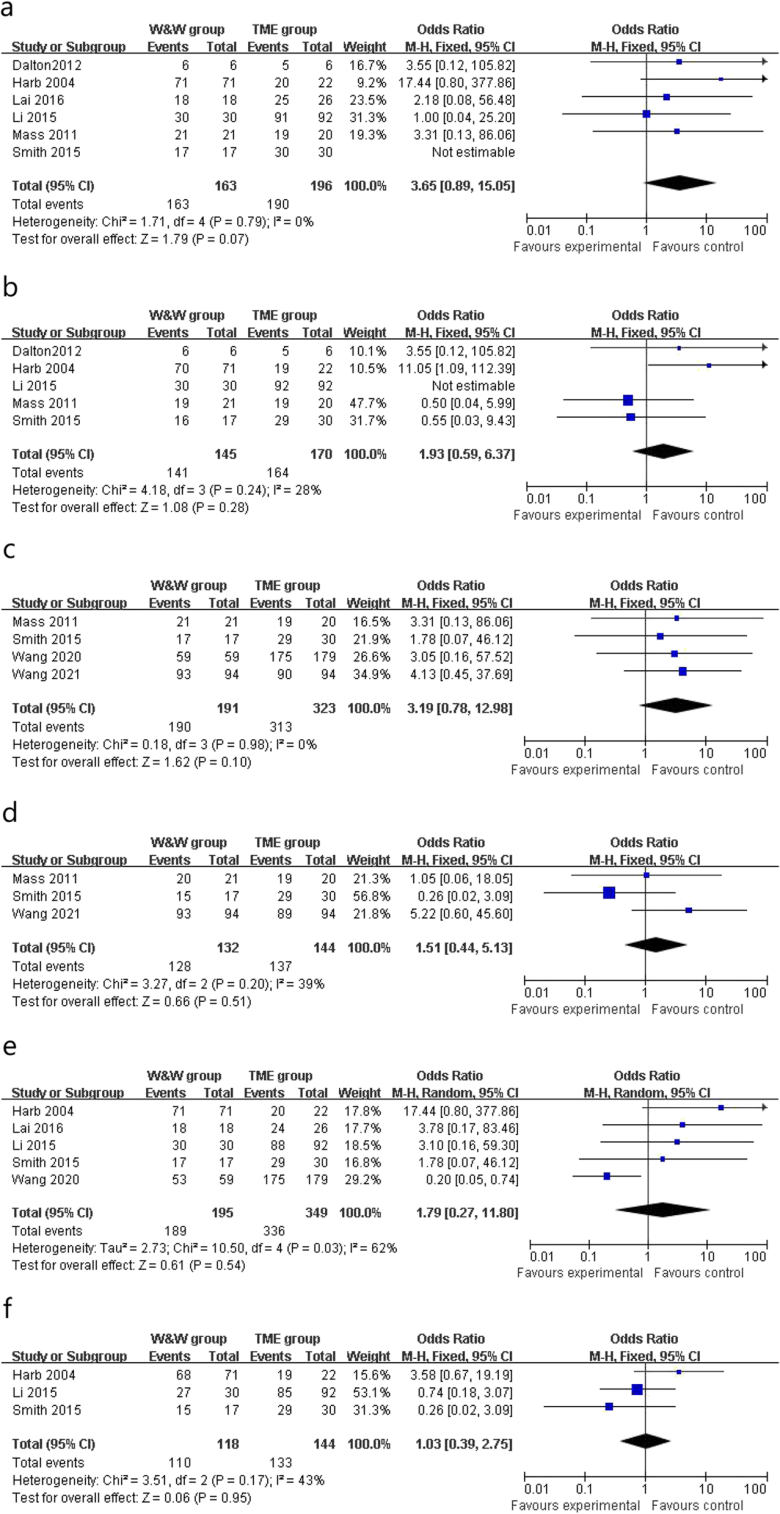
Outcomes of W&W group versus TME group. **a** 2-year DFS. **b** 2-year OS. **c** 3-year DFS. **d** 3-year OS. **e** 5-year DFS. **f** 5-year OS

The clinical data of 2-year OS were reported in 6 studies, the W&W group and the TME group had similar 2-year OS in the fixed-effects model with minimal heterogeneity (OR 3.65, 95% CI 0.89 to 15.05, *P* = 0.07, *χ*^2^ = 1.71, *P* = 0.79, *I*^2^ = 0%, 100.00% vs 98.43%, Fig. 3a). Five studies reported 2-year DFS and the two groups had similar 2-year DFS in the fixed-effects model with minimal heterogeneity (OR 1.93, 95% CI 0.59 to 6.37, *P* = 0.28, *χ*^2^ = 4.18, *P* = 0.24, *I*^2^ = 28%, 96.55% vs 96.47%, Fig. 3b). Three-year OS (OR 3.19, 95% CI 0.78 to 12.98, *P* = 0.10, *χ*^2^ = 0.18, *P* = 0.98, *I*^2^ = 0%, 99.47% vs 96.90%, Fig. 3c) and 3-year DFS (OR 1.51, 95% CI 0.44 to 5.13, *P* = 0.51, *χ*^2^ = 3.27, *P* = 0.20, *I*^2^ = 39%, 96.96% vs 95.14%, Fig. 3d) were similar in both groups in the fixed-effects model with minimal heterogeneity. In addition, 5-year OS (OR 1.79, 95% CI 0.27 to 11.80, *P* = 0.54, *χ*^2^ = 10.50, *P* = 0.03, *I*^2^ = 62%, 96.92% vs 96.28%, Fig. 3e) was similar in two groups in the random-effects model with significant heterogeneity, whereas 5-year DFS (OR 1.03, 95% CI 0.39 to 2.75, *P* = 0.95, *χ*^2^ = 3.51, *P* = 0.17, *I*^2^ = 43%, 93.22% vs 92.36%, Fig. 3f) was similar in two groups in the fixed-effects model with minimal heterogeneity.

### Publication bias

By using RevMan 5.0 and stata11.0 software, we used clinical recurrence data to detect publication bias. We obtained the funnel plot with the distributed points in the funnel plot. And the results of Egger’s test and Begg’s test indicated, there was no statistical difference for publication bias (*P* > 0.05). The details are shown in supplementary material 12.

## Discussion

According to NCCN guidelines, nCRT is the standard treatment for locally advanced rectal cancer. In 2004, Professor Habr-Gama first proposed the non-operative management (watch-and-wait strategy) for rectal cancer patients with a cCR response [30]. In 2012, Professor Mass proposed five diagnostic criteria for cCR. The more details were in the supplementary material 7 [21]. Nowadays, many studies have confirmed the therapeutic effect of the W&W strategy. TME surgery could bring huge trauma and serious postoperative complications to the patient. We summarized the results of postoperative complications. There was no surgical complication in the W&W group. But in the TME group, there were hernias in 7 patients, wound infections in 8 patients, intra-abdominal abscesses in 7 patients, fistula in 72 patients, and many other complications. Therefore, W&W strategy has no surgical trauma and can greatly improve the quality of life of patients.

### Novelty of the study

First, compared with the previous articles, the study included the latest studies with consistent baseline data. The results were similar to those of the previous articles (Zhao [31], Dossa [6], and Li [14] study). Second, we excluded studies with significantly different baseline data. Statistical differences in preoperative T staging, N staging and TNM staging could cause inconsistent baseline data and affect the final results. Third, the meta-analysis aimed to clarify salvage therapy and provide valuable information for the W&W strategy.

### Comparison with the previous research

The study of Zhao et al. [31] included 11 studies. He noted that the W&W group had a higher local recurrence rate than the TME group, and there was no significant difference in other outcomes [26–28, 31, 32]. There were 23 studies in the study of Dossa et al. [6], and they noted that the results (non-regrowth recurrence, cancer-specific mortality, OS, and DFS) were similar in the two groups. The study of Li et al. [14] included nine studies and noted that the non-surgical group had higher 1-, 2-, 3-, and 5-year local recurrence rates than the control groups and the other results were similar in the two groups. Our report further confirmed that the W&W group had a greater local recurrence rate than the TME group. With the help of physical examination and salvage therapy, W&W strategy could achieve similar treatment effects as the TME group, and this conclusion was consistent with that of Dattani et al. [33] [33–41]. The local recurrence rate of the W&W group was 21.6%, and 93% rectal cancer patients achieved R0 resection after local recurrence. The 3-year OS rate of the W&W group was 93.5%. The study of Dattani et al. [33] noted that robust surveillance with suitable salvage surgery makes the W&W strategy safe and effective. The study of Capelli et al. [42] included nine studies in his study, and he reported that the W&W strategy can also reduce the rate of colostomy in addition to the above conclusions [42]. The specific details are shown in supplementary material 13.

## Outcome results

### Local recurrence distant metastasis and cancer-related death

The local recurrence rate in the W&W group was greater than that in the TME group. We further confirmed the result (high local recurrence rate in the W&W group) of the study of Li et al. A total of 86.4% patients with T3–4 stage disease and 63.9% patients with N1–2 stage disease were included before nCRT, and some patients achieved cCR status with negative biopsy after nCRT. However, cCR did not mean no evidence of disease (NED), and some ypT1–3 patients could achieve cCR status. In the TME group, surgery removed the remaining lesions and lymph nodes and reduced the risk of local recurrence (9.97%). However, the opposite situation was noted in the W&W group, the remaining lesions could relapse again and increase the risk of local recurrence. Similar distant metastasis rates and cancer-related deaths of the two groups were also reported by Dossa [6] and Li [14]. Due to the substantial trauma and the postoperative complications of TME group, the W&W strategy had greatly reduced physical and psychological trauma, and ensured the quality of life while not increasing the probability of DM and CRD rate.

### DFS and OS

The 2-year OS and 2-year DFS in the W&W group were 100% and 96.5% respectively. The 2-year OS and 2-year DFS in the TME group were 98.4% and 96.4% respectively. The 3-year OS, 3-year DFS, 5-year OS and 5-year DFS were similar in the two groups. Initially, we analyzed the studies (including the studies with significant differences in baseline data, such as Lin, Yeom, Lee study) with RevMan 5.0 and 2-DFS in the W&W group was better than that in the TME group. The specific information is shown in the supplementary material 14 [43]. We hypothesized that the large difference in the baseline data and the incomplete information of Lin, Yeom, Lee, and other studies could affect the results of the research, so we only included studies with consistent baseline data. The similar DFS and OS in the two groups were consistent with previous researches. Patients with cCR status had a good response to NCRT, indicating that the tumor had good biological behavior and that the patients with cCR status could experience a long survival time in the two groups. Ayloor [16] and Smith [22] reported that the tumor recurrence time was mainly 12 months. Lai [19], Li [20], and Mass [21] reported that the tumor recurrence time was mainly 24 months. We speculated that fewer patients relapsed and did not reach a statistical difference, so the DFS was similar in the two groups. With the help of physical examination and salvage surgery, the patient received the corresponding treatment after tumor relapse and the mean overall survival (months) of patients who relapsed also reached more than 50 months. So, the treatment effect of salvage surgery was reliable.

### nCRT plan and salvage therapy for local recurrence

The currently nCRT plan is intensity-modulated radiotherapy (45–50.4 Gy/25–28f) with oral sensitizer (capecitabine 825 mg/m^2^/bid po). Surgery could be performed eight weeks after nCRT, and the tumour could be minimized to increase the anus preservation rate and pCR rate. Marit et al. [44] reported that 85 (22.1%) out of 385 rectal cancer cCR patients relapsed after a median of nine months. Eighty-four (98.8%) patients underwent salvage surgery, 58 (69%) patients underwent TME, and 26 (30.6%) patients underwent local excision. The 2-DFS and 2-OS of patients who underwent salvage surgery were 90.3% and 98.4% respectively [44]. Irfan [45] et al. proposed that the anastomotic leaks, 30-day morbidity and reintervention rate were similar in the non-deferred surgery group and regrowth deferred surgery group [45]. Simpson [46] et al. noted that the W&W strategy was safe in this patient cohort, with acceptable rates of local regrowth and survival [46]. In our study, 30 (83.33%) patients with local recurrence in the W&W group underwent the salvage therapy. TME surgery was the main type of salvage therapy and could prolong OS. Therefore, with the help of physical examination and salvage treatment, the W&W strategy could achieve good clinical results with low hospitalization costs [47].

## Limitations

This study might have several limitations. First, no RCTs were available, and the included studies and patients were limited. It could cause bias and affect the results. Second, different levels of medical technology in distinct areas and the partially missing clinical data could affect the final results. Thirdly, the relevant data of adjuvant chemotherapy were incomplete; we were unable to describe the specific plan and treatment effect of adjuvant chemotherapy. It could be an important confounding factor in the TME group following surgery. Finally, minimal information on T stage and N stage could lead to limited conclusions regarding specific treatment methods for the patients. Hence, we hope that more RCTs on this topic will emerge in the future.

## Conclusion

The meta-analysis used the latest data to compare the advantages and disadvantages of the W&W strategy and TME surgery for locally advanced rectal cancer with cCR status after nCRT. The W&W group had a higher risk of local recurrence than the TME group, but a similar OS was observed in the two groups. With the help of physical examination and salvage treatment, the W&W strategy could not only decrease surgical trauma and ensure quality of life, but also achieve good clinical results. Hence, the W&W strategy could represent a beneficial model for rectal cancer with cCR status.

## Supplementary Information

**Additional file 1.**

**Additional file 2.** Systematic review.

**Additional file 3.** PRISMA 2009 Checklist.

**Additional file 4.** The details of PICOS.

**Additional file 5.** The information of the search terms.

**Additional file 6.** The details of neoadjuvant treatment of studies.

**Additional file 7.** The details of diagnostic criteria (methods) and surveillance strategies of each method in each study.

**Additional file 8.** Characteristics of the included articles.

**Additional file 9.** The details of salvage therapy.

**Additional file 10.** The details of salvage therapy in W&W group.

**Additional file 11.** The complications of salvage therapy in W&W group and TME group.

**Additional file 12.**

**Additional file 13.** Comparison between the previous researches.

**Additional file 14.**

**Additional file 15.** Editing certificate. A meta-analysis of the watch-and-wait strategy versus total mesorectal excision for rectal cancer exhibiting complete clinical response after.

**Additional file 16.** Editing certificate. A meta-analysis of watch and wait strategy versus total mesorectal excision for rectal cancer with clinical complete response after neoadjuvant chemoradiotherapy.

**Additional file 17: Fig 1.** Flowchart of the included studies. **Fig 2.** Outcomes of W&W group versus TME group. a. local recurrence; b: distant metastasis; c: cancer related death. **Fig 3.** Outcomes of W&W group versus TME group. a. 2-year DFS; b: 2-year OS; c: 3-year DFS; d: 3-year OS; e: 5-year DFS; f: 5-year OS.

**Additional file 18.** Statistical method.

**Additional file 19.** the details of adjuvant chemotherapy of studies.

**Additional file 20.** Search terms and database.

## Abbreviations

PNCT: Prospective non-randomized controlled trial
RNCT: Retrospective non-randomized controlled trial
cCR: Complete clinical response
nCRT: Neoadjuvant chemoradiotherapy
5-FU: 5-Fluorouracil
Cape: Capecitabine
TME: Total mesorectal excision
APR: Abdominal-perineal resection
LAR: Low anterior resection
CI: Confidence interval
OR: Odds ratios
OS: Overall survival
SMD: Standardized mean difference
