# Supplementary material for: A meta-analysis of the watch-and-wait strategy versus total mesorectal excision for rectal cancer exhibiting complete clinical response after neoadjuvant chemoradiotherapy
Source: World J Surg Oncol. 2021 Oct 18;19:305. doi: 10.1186/s12957-021-02415-y (PMC8522111; doi:10.1186/s12957-021-02415-y)

https://www.crd.york.ac.uk/prospero/#myprospero
https://www.crd.york.ac.uk/prospero/#recordDetails

ID：CRD42021244032

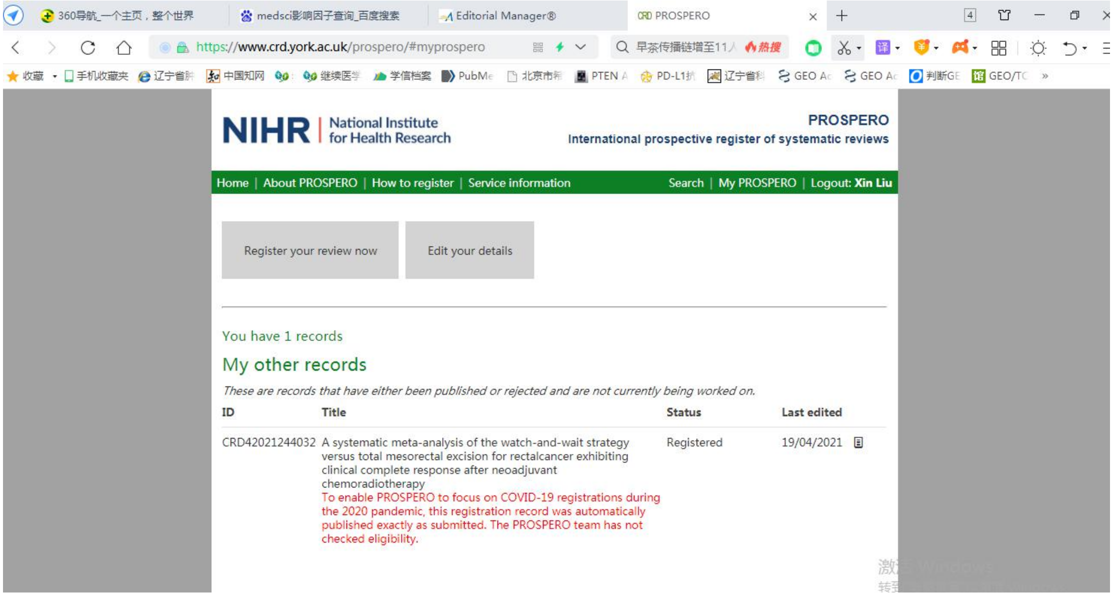

Supplement: Supplementary file 1 — Additional file 1. [file 12957_2021_2415_MOESM1_ESM.pdf]
