# Supplementary material for: A meta-analysis of the watch-and-wait strategy versus total mesorectal excision for rectal cancer exhibiting complete clinical response after neoadjuvant chemoradiotherapy
Source: World J Surg Oncol. 2021 Oct 18;19:305. doi: 10.1186/s12957-021-02415-y (PMC8522111; doi:10.1186/s12957-021-02415-y)
Supplement: Supplementary file 2 — Additional file 2. Systematic review. [file 12957_2021_2415_MOESM2_ESM.pdf]

National Institute for Health Research

UNIVERSITY of York
Centre for Reviews and Dissemination

Systematic review

1. * Review title.

Give the title of the review in English

A systematic meta-analysis of the watch-and-wait strategy versus total mesorectal excision for rectalcancer exhibiting clinical complete response after neoadjuvant chemoradiotherapy

2. Original language title.

For reviews in languages other than English, give the title in the original language. This will be displayed with the English language title.

Meta-analysis of the watch-and-wait strategy versus total mesorectal excision for rectalcancer exhibiting clinical complete response after neoadjuvant chemoradiotherapy

3. * Anticipated or actual start date.

Give the date the systematic review started or is expected to start.

31/03/2021

4. * Anticipated completion date.

Give the date by which the review is expected to be completed.

31/08/2022

5. * Stage of review at time of this submission.

Tick the boxes to show which review tasks have been started and which have been completed. Update this field each time any amendments are made to a published record.

Reviews that have started data extraction (at the time of initial submission) are not eligible for inclusion in PROSPERO. If there is later evidence that incorrect status and/or completion date has been supplied, the published PROSPERO record will be marked as retracted.

This field uses answers to initial screening questions. It cannot be edited until after registration.

The review has not yet started: No

Review stage	Started	Completed
Preliminary searches	Yes	No
Piloting of the study selection process	Yes	No
Formal screening of search results against eligibility criteria	No	No
Data extraction	No	No
Risk of bias (quality) assessment	No	No
Data analysis	No	No

Provide any other relevant information about the stage of the review here.

6. * Named contact.

The named contact is the guarantor for the accuracy of the information in the register record. This may be any member of the review team.

Xin Liu

Email salutation (e.g. "Dr Smith" or "Joanne") for correspondence:

Mr Liu

7. * Named contact email.

Give the electronic email address of the named contact.

liuxin5626855@sina.com

8. Named contact address

Give the full institutional/organisational postal address for the named contact.

Cancer hospital of China medical university, Liaoning cancer hospital and institute\nshenyang, xiao he yan road, No.44, Liaoning Province CHINA

9. Named contact phone number.

Give the telephone number for the named contact, including international dialling code.

8618900918981

10. * Organisational affiliation of the review.

Full title of the organisational affiliations for this review and website address if available. This field may be completed as 'None' if the review is not affiliated to any organisation.

Cancer Hospital of China Medical University. Liaoning Cancer Hospital and Institute

Organisation web address:

http://www.lnszl.com/

11. * Review team members and their organisational affiliations.

Give the personal details and the organisational affiliations of each member of the review team. Affiliation refers to groups or organisations to which review team members belong. **NOTE: email and country now MUST be entered for each person, unless you are amending a published record.**

Mr Xin Liu. Cancer Hospital of China Medical University. Liaoning Cancer Hospital and Institute
undefined undefined undefined. undefined
Mr Zhouguang Jiao. Institute of Process Engineering, Chinese Academy of Science,
Mr Shiyang Ma. Department of Colorectal surgery, Cancer Hospital of China Medical University. Liaoning Cancer Hospital and Institute
Miss Wenqing Lu. School of Life Sciences, Hebei University
Mr Jun Qiao. Department of Colorectal surgery, Cancer Hospital of China Medical University. Liaoning Cancer Hospital and Institute
Mr Rui Zhang. Department of Colorectal surgery, Cancer Hospital of China Medical University. Liaoning Cancer Hospital and Institute

12. * Funding sources/sponsors.

Details of the individuals, organizations, groups, companies or other legal entities who have funded or sponsored the review.

No

Grant number(s)

State the funder, grant or award number and the date of award

13. * Conflicts of interest.

List actual or perceived conflicts of interest (financial or academic).

None

14. Collaborators.

Give the name and affiliation of any individuals or organisations who are working on the review but who are not listed as review team members. **NOTE: email and country must be completed for each person, unless you are amending a published record.**

15. * Review question.

State the review question(s) clearly and precisely. It may be appropriate to break very broad questions down into a series of related more specific questions. Questions may be framed or refined using PI(E)COS or similar where relevant.

Whether watch-and-wait strategy was suitable for rectal cancer exhibiting clinical complete response after neoadjuvant chemoradiotherapy? In our meta-analysis, we focused on the stage I to III rectal cancer with clinical complete response after neoadjuvant chemoradiotherapy, and we tried to include more studies with no difference in the baseline data to assess the advantages and disadvantages between watch-and-wait group and total mesorectal excision group.

16. * Searches.

State the sources that will be searched (e.g. Medline). Give the search dates, and any restrictions (e.g. language or publication date). Do NOT enter the full search strategy (it may be provided as a link or

attachment below.)

We will search, with no time restrictions, the following databases for relevant English language literature: PubMed, Cochrane Library, CNKI(China National Knowledge Infrastructure) and Wanfang databases to obtain relevant literature (up to January 2021). The search string will be built as follows:“watch-and-wait” or “nonoperative management” or “total mesorectal excision” or “neoadjuvant chemoradiotherapy” and “rectal cancer”. The electronic database search will be supplemented by a manual search.

17. URL to search strategy.

Upload a file with your search strategy, or an example of a search strategy for a specific database, (including the keywords) in pdf or word format. In doing so you are consenting to the file being made publicly accessible. Or provide a URL or link to the strategy. Do NOT provide links to your search **results**.

https://PubMed.ncbi.nlm.nih.gov/?term=%28%28%28%28watch-and-wait%29+OR+%28nonoperative+management%29%29+OR+%28total+mesorectal+excision%29%29+OR+%28neoadjuvant+chemoradiotherapy%29%29+AND+%28rectal+cancer%29&sort=

Alternatively, upload your search strategy to CRD in pdf format. Please note that by doing so you are consenting to the file being made publicly accessible.

Do not make this file publicly available until the review is complete

18. * Condition or domain being studied.

Give a short description of the disease, condition or healthcare domain being studied in your systematic review.

Given its high incidence and fatality rate, rectal cancer seriously endangers human health. In particular, some patients with locally advanced rectal cancer would have their anus removed, which could cause substanial trauma to the patients. Neoadjuvant chemoradiotherapy (nCRT) combined with surgical treatment has become the standard treatment mode for the locally advanced rectal cancer. nCRT could reduce the local recurrence of patients with locally advanced rectal cancer.Multiple colorectal cancer guidelines recommend total mesorectal excision (TME) after nCRT as the standard for locally advanced rectal cancer, but TME surgery itself has various complications, such as bleeding, intestinal obstruction, anastomotic leakage and other complications. The tumours of some patients could disappear completely after neoadjuvant chemoradiotherapy, and the specific phenomenon is defined as a clinical complete response(cCR). In 2004, Professor Habr-Gama proposed that patients with cCR status could adopt the watch-and-wait (W&W) treatment strategy. Since then, a series of clinical research reports have promoted the discussion about the treatment strategy of cCR patients. Compared with TME surgery, the W&W strategy could achieve a similar overall survival rate and a better outcome of preservation of organ anatomy and physiological function.

19. * Participants/population.

Specify the participants or populations being studied in the review. The preferred format includes details of both inclusion and exclusion criteria.

Inclusion criteria: the study had consistent baseline database and the available clinical data between the two

groups; total mesorectal excision included APR (abdominal-perineal resection), Dixon and other radical surgical approaches, but not local excision; rectal cancer patients (stage I to III) with cCR status after nCRT.

Exclusion criteria: rectal cancer patients did not achieve cCR status after nCRT

20. * Intervention(s), exposure(s).

Give full and clear descriptions or definitions of the interventions or the exposures to be reviewed. The preferred format includes details of both inclusion and exclusion criteria.

Watch-and-wait strategy was the main intervention.

21. * Comparator(s)/control.

Where relevant, give details of the alternatives against which the intervention/exposure will be compared (e.g. another intervention or a non-exposed control group). The preferred format includes details of both inclusion and exclusion criteria.

Total mesorectal excision was the main comparator.

22. * Types of study to be included.

Give details of the study designs (e.g. RCT) that are eligible for inclusion in the review. The preferred format includes both inclusion and exclusion criteria. If there are no restrictions on the types of study, this should be stated.

RCTs (Randomized Controlled Trial), RCNTs (retrospective comparative non-randomized studies), PCNTs (prospective comparative non-randomized studies), cohort studies or case-control studies

23. Context.

Give summary details of the setting or other relevant characteristics, which help define the inclusion or exclusion criteria.

24. * Main outcome(s).

Give the pre-specified main (most important) outcomes of the review, including details of how the outcome is defined and measured and when these measurement are made, if these are part of the review inclusion criteria.

Local recurrence(LE), cancer-related death (CRD) and distant metastasis (DM) were the primary outcomes of the study.

Measures of effect

Please specify the effect measure(s) for you main outcome(s) e.g. relative risks, odds ratios, risk difference, and/or 'number needed to treat.

OR/RR

25. * Additional outcome(s).

List the pre-specified additional outcomes of the review, with a similar level of detail to that required for main outcomes. Where there are no additional outcomes please state ‘None’ or ‘Not applicable’ as appropriate to the review

2-, 3- and 5- year disease-free survival (DFS) and overall survival (OS) were the secondary outcomes.

Measures of effect

Please specify the effect measure(s) for you additional outcome(s) e.g. relative risks, odds ratios, risk difference, and/or 'number needed to treat.

OR/RR

26. * Data extraction (selection and coding).

Describe how studies will be selected for inclusion. State what data will be extracted or obtained. State how this will be done and recorded.

The clinically useful data were collected by two reviewers independently according to the Newcastle-Ottawa Scale (NOS) guidelines. Any disagreement would be resolved by discussion until consensus was reached or by consulting a third author.

27. * Risk of bias (quality) assessment.

State which characteristics of the studies will be assessed and/or any formal risk of bias/quality assessment tools that will be used.

By using RevMan 5.0 software, we made use of the local recurrence rate to detect publication bias.

28. * Strategy for data synthesis.

Describe the methods you plan to use to synthesise data. This **must not be generic text** but should be **specific to your review** and describe how the proposed approach will be applied to your data. If meta-analysis is planned, describe the models to be used, methods to explore statistical heterogeneity, and software package to be used.

We performed the meta-analysis by using RevMan 5.0 and Stata 11.0 software. Continuous data and dichotomous data were evaluated by the standardized mean differences (SMDs) and relative risks (ORs or RRs) with 95% confidence intervals respectively. We used the I^2 statistic and funnel plots to assess the heterogeneity and publication bias separately. We used random effects models to analyse, the data with huge heterogeneity (I^2?50%) and the fixed effects model for little heterogeneity ($I^2$50%).

29. * Analysis of subgroups or subsets.

State any planned investigation of ‘subgroups’. Be clear and specific about which type of study or participant will be included in each group or covariate investigated. State the planned analytic approach.

No subgroup

30. * Type and method of review.

Select the type of review, review method and health area from the lists below.

Type of review

Cost effectiveness
No

Diagnostic
No

Epidemiologic
No

Individual patient data (IPD) meta-analysis
No

Intervention
Yes

Living systematic review
No

Meta-analysis
Yes

Methodology
No

Narrative synthesis
No

Network meta-analysis
No

Pre-clinical
No

Prevention
No

Prognostic
No

Prospective meta-analysis (PMA)
No

Review of reviews
No

Service delivery
No

Synthesis of qualitative studies
No

Systematic review
Yes

Other
No

Health area of the review

Alcohol/substance misuse/abuse
No

Blood and immune system
No

Cancer
Yes

Cardiovascular
No

Care of the elderly
No

Child health
No

Complementary therapies
No

COVID-19
No

Crime and justice
No

Dental
No

Digestive system
Yes

Ear, nose and throat
No

Education
No

Endocrine and metabolic disorders
No

Eye disorders
No

General interest
No

Genetics
No

Health inequalities/health equity
No

Infections and infestations
No

International development
No

Mental health and behavioural conditions
No

Musculoskeletal
No

Neurological
No

Nursing
No

Obstetrics and gynaecology
No

Oral health
No

Palliative care
No

Perioperative care
No

Physiotherapy
No

Pregnancy and childbirth
No

Public health (including social determinants of health)
No

Rehabilitation
No

Respiratory disorders
No

Service delivery
No

Skin disorders
No

Social care
No

Surgery
No

Tropical Medicine
No

Urological
No

Wounds, injuries and accidents
No

Violence and abuse
No

31. Language.

Select each language individually to add it to the list below, use the bin icon to remove any added in error.
English

There is not an English language summary

32. * Country.

Select the country in which the review is being carried out. For multi-national collaborations select all the countries involved.

China

33. Other registration details.

Name any other organisation where the systematic review title or protocol is registered (e.g. Campbell, or The Joanna Briggs Institute) together with any unique identification number assigned by them. If extracted data will be stored and made available through a repository such as the Systematic Review Data Repository

(SRDR), details and a link should be included here. If none, leave blank.

No

34. Reference and/or URL for published protocol.

If the protocol for this review is published provide details (authors, title and journal details, preferably in Vancouver format)

No

Add web link to the published protocol.

Or, upload your published protocol here in pdf format. Note that the upload will be publicly accessible.

No I do not make this file publicly available until the review is complete

Please note that the information required in the PROSPERO registration form must be completed in full even if access to a protocol is given.

35. Dissemination plans.

Do you intend to publish the review on completion?

No

Give brief details of plans for communicating review findings.?

36. Keywords.

Give words or phrases that best describe the review. Separate keywords with a semicolon or new line. Keywords help PROSPERO users find your review (keywords do not appear in the public record but are included in searches). Be as specific and precise as possible. Avoid acronyms and abbreviations unless these are in wide use.

watch-and-wait, complete clinical response, total mesorectal excision, meta-analysis.

37. Details of any existing review of the same topic by the same authors.

If you are registering an update of an existing review give details of the earlier versions and include a full bibliographic reference, if available.

No

38. * Current review status.

Update review status when the review is completed and when it is published.New registrations must be ongoing so this field is not editable for initial submission.
Please provide anticipated publication date

Review_Completed_published

39. Any additional information.

Provide any other information relevant to the registration of this review.

No

40. Details of final report/publication(s) or preprints if available.

Leave empty until publication details are available OR you have a link to a preprint (NOTE: this field is not

editable for initial submission). List authors, title and journal details preferably in Vancouver format.

No

Give the link to the published review or preprint.
