# Supplementary material for: A meta-analysis of the watch-and-wait strategy versus total mesorectal excision for rectal cancer exhibiting complete clinical response after neoadjuvant chemoradiotherapy
Source: World J Surg Oncol. 2021 Oct 18;19:305. doi: 10.1186/s12957-021-02415-y (PMC8522111; doi:10.1186/s12957-021-02415-y)
Supplement: Supplementary file 4 — Additional file 4. The details of PICOS. [file 12957_2021_2415_MOESM4_ESM.doc]

Supplementary material 4: The details of PICOS

|  | The details of PICOS |
| --- | --- |
| P (Population) | Rectal cancer patients with cCR response after neoadjuvant chemoradiotherapy |
| I (Intervention ) | watch-and-wait strategy |
| C (Comparator) | total mesorectal excision |
| O (Outcomes) | Primary outcomes:  Local recurrence(LR): endoscopic pathology or other examination to confirm that the tumor that has regressed reappears  Distant metastasis(DM): the absence of metastases diagnosed by CT pathology or other methods  Cancer related death(CRD): The patient is related to death caused by cancer  Secondary outcomes:  Disease-free survival(DFS): the absence of local or distant recurrence and death from any cause  Overall survival(OS) the absence of death from any cause |
