# Supplementary material for: A meta-analysis of the watch-and-wait strategy versus total mesorectal excision for rectal cancer exhibiting complete clinical response after neoadjuvant chemoradiotherapy
Source: World J Surg Oncol. 2021 Oct 18;19:305. doi: 10.1186/s12957-021-02415-y (PMC8522111; doi:10.1186/s12957-021-02415-y)

**Supplementary material 5: the information of the search terms.**

**Pubmed**

("watch-and-wait"[All Fields] OR (("nonop"[All Fields] OR "nonoperative"[All Fields] OR "nonoperatively"[All Fields]) AND ("manage"[All Fields] OR "managed"[All Fields] OR "management s"[All Fields] OR "managements"[All Fields] OR "manager"[All Fields] OR "manager s"[All Fields] OR "managers"[All Fields] OR "manages"[All Fields] OR "managing"[All Fields] OR "managment"[All Fields] OR "organization and administration"[MeSH Terms] OR ("organization"[All Fields] AND "administration"[All Fields]) OR "organization and administration"[All Fields] OR "management"[All Fields] OR "disease management"[MeSH Terms] OR ("disease"[All Fields] AND "management"[All Fields]) OR "disease management"[All Fields])) OR (("total"[All Fields] OR "totaled"[All Fields] OR "totaling"[All Fields] OR "totalled"[All Fields] OR "totalling"[All Fields] OR "totals"[All Fields]) AND "mesorectal"[All Fields] AND ("excisable"[All Fields] OR "excise"[All Fields] OR "excised"[All Fields] OR "excises"[All Fields] OR "excising"[All Fields] OR "excision"[All Fields] OR "excisions"[All Fields])) OR ("neoadjuvant therapy"[MeSH Terms] OR ("neoadjuvant"[All Fields] AND "therapy"[All Fields]) OR "neoadjuvant therapy"[All Fields] OR ("neoadjuvant"[All Fields] AND "chemoradiotherapy"[All Fields]) OR "neoadjuvant chemoradiotherapy"[All Fields])) AND ("rectal neoplasms"[MeSH Terms] OR ("rectal"[All Fields] AND "neoplasms"[All Fields]) OR "rectal neoplasms"[All Fields] OR ("rectal"[All Fields] AND "cancer"[All Fields]) OR "rectal cancer"[All Fields])


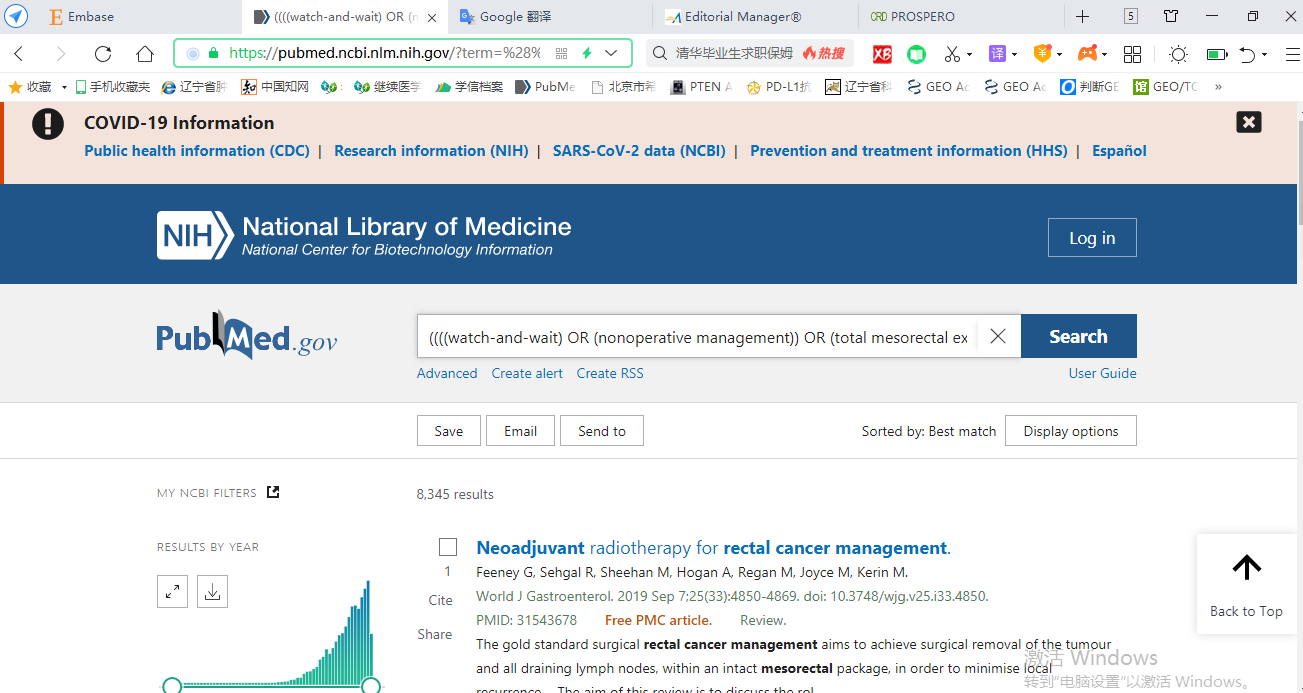


**EMBASE database**

search terms:(watch AND wait OR (nonoperative AND management) OR (total AND mesorectal AND excision) OR (neoadjuvant AND chemoradiotherapy)) AND ('rectal cancer'/exp OR 'rectal cancer' OR (rectal AND ('cancer'/exp OR cancer)))


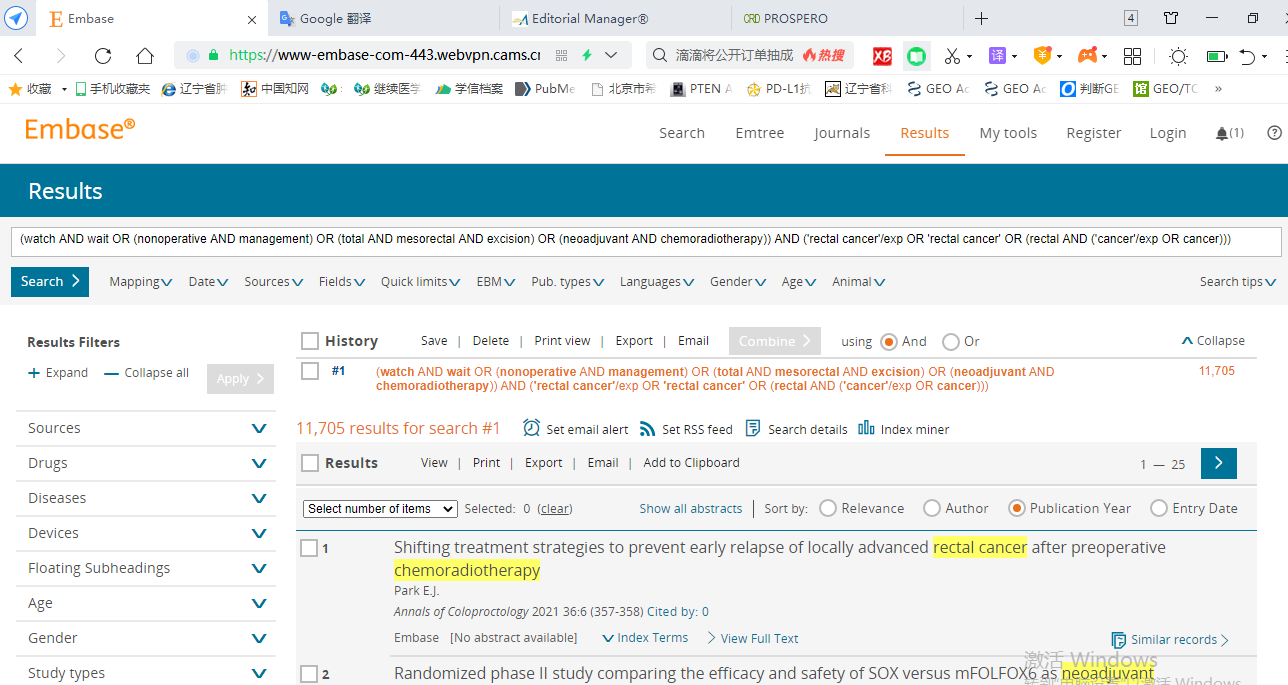


**Other databases did not have the detailed search terms similar to Pubmed, so I will describe the search terms and the search results in the following text.**

**Cochrane library**

362 Cochrane Reviews, 56 Cochrane Protocols matching, 3376 Trials, 1 Special Collection, 9 Clinical Answers matching watch and wait in All Text OR nonoperative management in All Text OR total mesorectal excision in All Text OR neoadjuvant chemoradiotherapy in All Text AND rectal cancer in All Text - (Word variations have been searched)


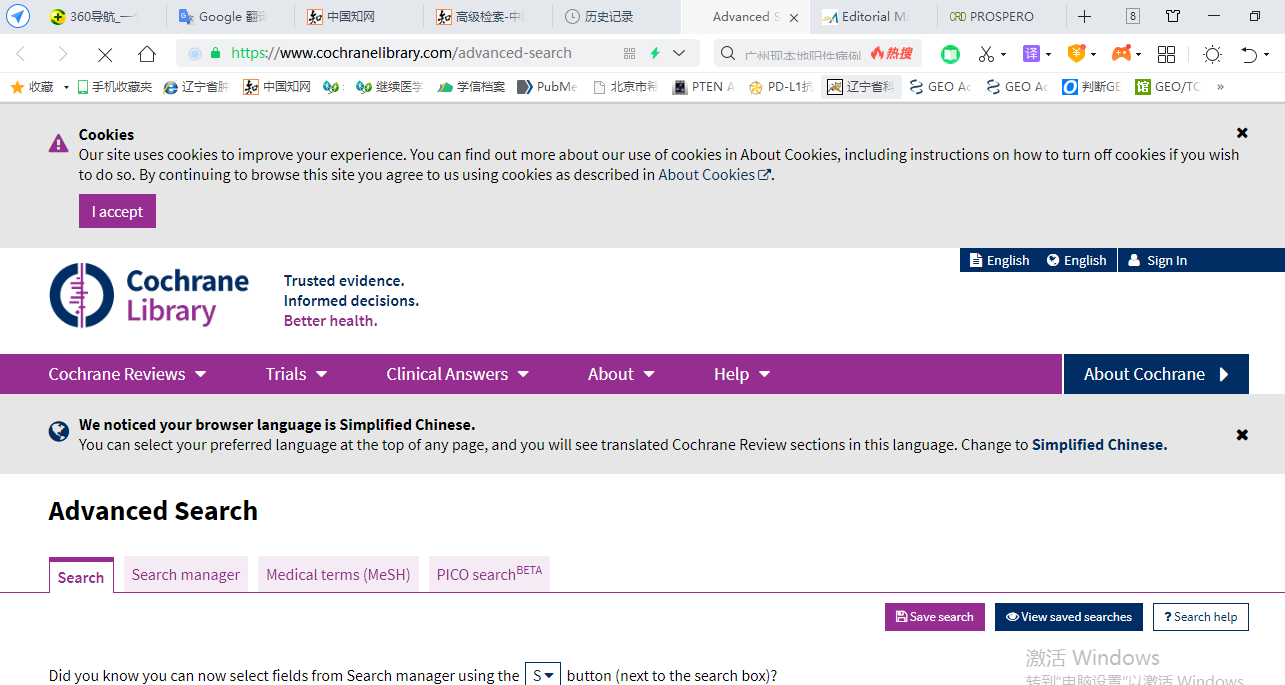


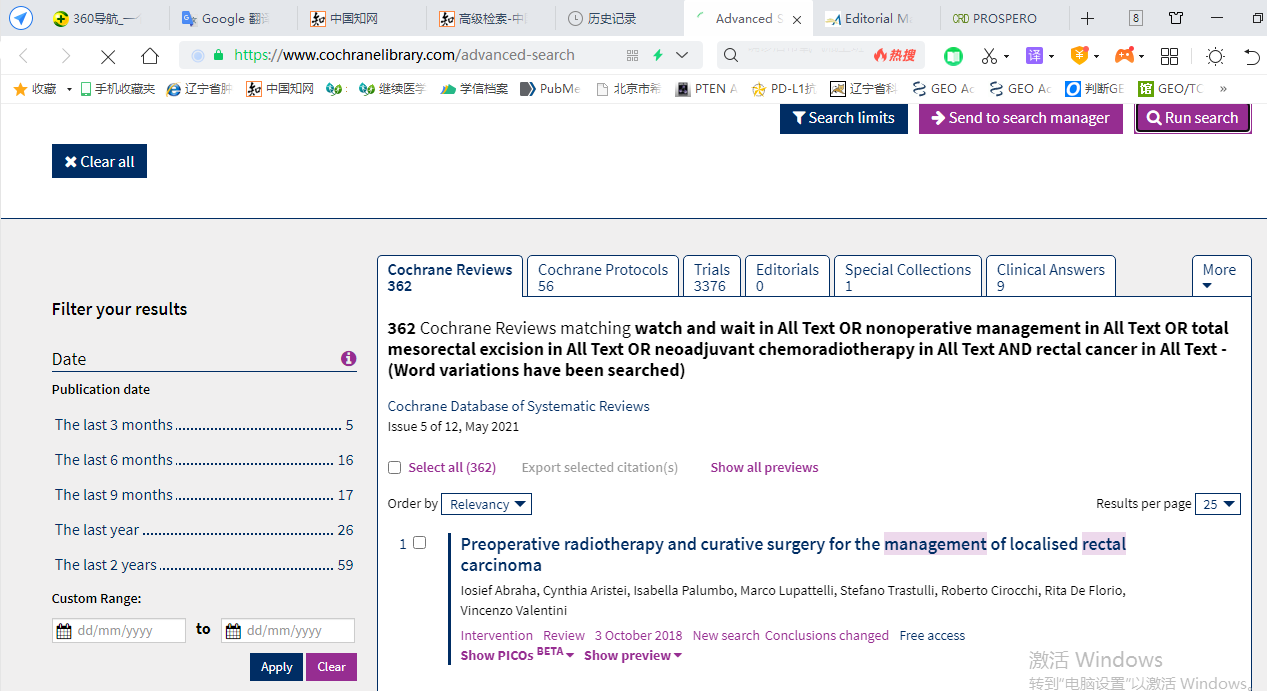


**Wangfang database**

search terms: all: (watchful waiting) or all: (non-surgical treatment) or all: (total mesangial resection) or all: (neoadjuvant therapy) and all: (rectal cancer)


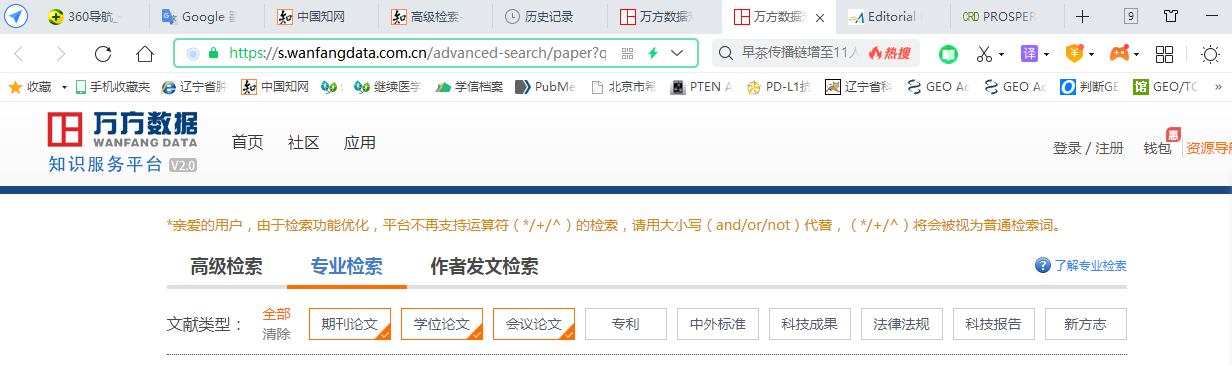

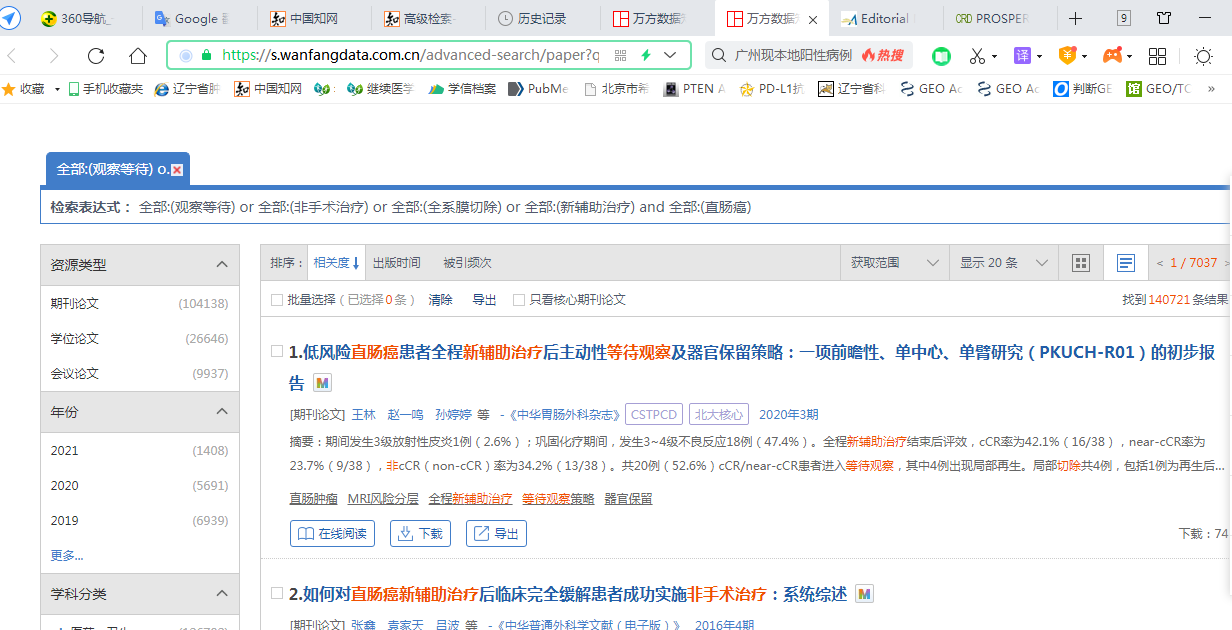


**CNKI database**

search terms: (Full text: watchful waiting) OR (full text: non-surgical treatment) OR (full text: total mesangial resection) OR (full text: neoadjuvant treatment) AND (full text: rectal cancer)


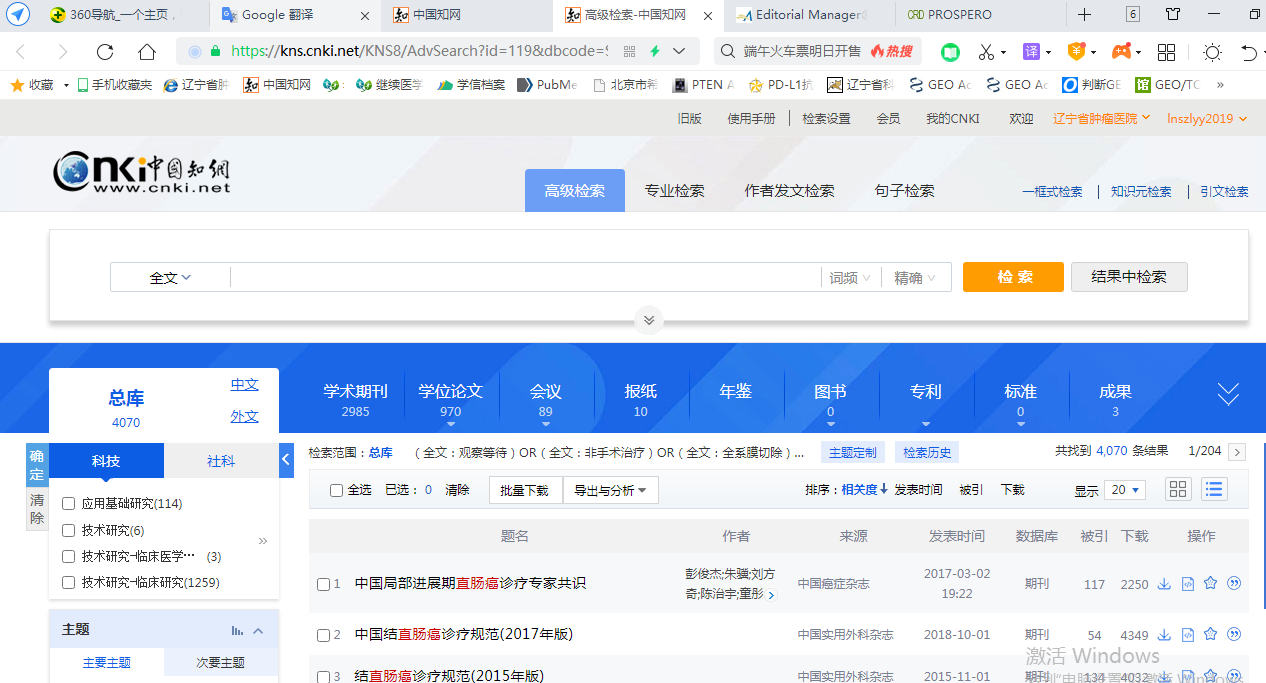

Supplement: Supplementary file 5 — Additional file 5. The information of the search terms. [file 12957_2021_2415_MOESM5_ESM.doc]
