# Supplementary material for: A meta-analysis of the watch-and-wait strategy versus total mesorectal excision for rectal cancer exhibiting complete clinical response after neoadjuvant chemoradiotherapy
Source: World J Surg Oncol. 2021 Oct 18;19:305. doi: 10.1186/s12957-021-02415-y (PMC8522111; doi:10.1186/s12957-021-02415-y)
Supplement: Supplementary file 6 — Additional file 6. The details of neoadjuvant treatment of studies. [file 12957_2021_2415_MOESM6_ESM.doc]

**Supplementary material 6: the details of neoadjuvant treatment of studies**

| Study | Neoadjuvant therapy | Evaluation time before surgery (week) | PCR(%) |
| --- | --- | --- | --- |
| Ayloor[16] | NCRT (Neoadjuvant chemoradiation). The specific plan is unknown. | The assessment is undertaken at 4-6 weeks to evaluate the tumor. | 30 |
| Dalton[17] | 45 Gy in 25 fractions over 5 weeks with concurrent Capecitabine (825 mg⁄m^2^). | A repeat MRI scan is undertaken at 6 to 8 weeks to evaluate the tumor. | 16.6 |
| Habr[18] | 50.40 Gy delivered at the isodose line given at 180 cGy/d for 5 days per week, for 6 consecutive weeks, using a 6-mV to 18-mV linear accelerator. 5-fluoracil (425 mg/m^2^/d) and folinic acid (20 mg/m^2^/d) administered intravenously for 3 consecutive days on the first and last 3 days of radiation therapy. | The assessment is undertaken at 8 weeks to evaluate the tumor. | 8.3 |
| Lai[19] | 5-FU was administered as a bolus (350 mg/m^2^/day) with a low-dose leucovorin bolus (10 mg/m^2^/day) for 5 days on days 1–5 and 29–33 in combination with radiotherapy (45 Gy in 25 fractions or 54 Gy in 30 fractions). | The assessment is undertaken at 8-12 weeks to evaluate the tumor. | - |
| Li[20] | NCRT (50 Gy/25 f/2 Gy, capecitabine, 825 mg/m^2^ bid, concurrently). | The assessment is undertaken at 8-10 weeks to evaluate the tumor. | 88 |
| Mass[21] | CRT consisted of 28 fractions of 1.8 Gy combined with 2*825 mg/m^2^ capecitabine. | The assessment is undertaken at 6-8 weeks to evaluate the tumor. | - |
| Smith[22] | The median dose of radiotherapy administered was 50.40 Gy (range 45–56 Gy). plus 5-flfluorouracil (5-FU) or capecitabine. The specific plan is unknown. | The assessment is undertaken at 4-10 weeks to evaluate the tumor. | - |
| Wang[23] | CRT consisted of GTV 50 Gy/25 fractions /5 weeks, CTV 45-46 Gy/ fractions /5 weeks combined with 2-4 cycles 5-fluorouracil or capecitabin based chemotherapy. The specific plan is unknown. | The assessment is undertaken at 6-12 weeks to evaluate the tumor. | - |
| Wang[24] | quently with a total dose of 50 Gy. All patients received fuorouracil based chemoradiotherapy. A total of 68 (72.3%) patients in the watch-and-wait group received induction and/or consolidation chemotherapy, while 84 (89.4%) patients in the surgical group received perioperative chemotherapy in addition to chemoradiotherapy. | The assessment is undertaken at 6-12 weeks to evaluate the tumor. | 51.1 |

TME: total mesorectal excision; APR: abdominal-perineal resection;; LAR: Low anterior resection; CAA:coloanal anastomosis

NCRT neoadjuvant chemoradiotherapy; NR:no record.
