# Supplementary material for: A meta-analysis of the watch-and-wait strategy versus total mesorectal excision for rectal cancer exhibiting complete clinical response after neoadjuvant chemoradiotherapy
Source: World J Surg Oncol. 2021 Oct 18;19:305. doi: 10.1186/s12957-021-02415-y (PMC8522111; doi:10.1186/s12957-021-02415-y)
Supplement: Supplementary file 8 — Additional file 8. Characteristics of the included articles. [file 12957_2021_2415_MOESM8_ESM.doc]

**Supplementary material 8: characteristics of the included articles**

| Study | BMI | | Diabetes mellitus | | Hypertension | |
| --- | --- | --- | --- | --- | --- | --- |
|  | W&W | TME | W&W | TME | W&W | TME |
| Ayloor[16] | NR | NR | NR | NR | NR | NR |
| Dalton[17] | NR | NR | NR | NR | NR | NR |
| Habr[18] | NR | NR | NR | NR | NR | NR |
| Lai[19] | NR | NR | NR | NR | NR | NR |
| Li[20] | NR | NR | NR | NR | NR | NR |
| Mass[21] | NR | NR | NR | NR | NR | NR |
| Smith[22] | NR | NR | 1 (5.6 %) | 6 (20.0 %) | NR | NR |
| Wang[23] | NR | NR | NR | NR | NR | NR |
| Wang[24] | NR | NR | NR | NR | NR | NR |

| Study | Radical surgery type | | | | | Pre-NCRT CEA(ng/ml) | | Post-CRT CEA(ng/ml) | |
| --- | --- | --- | --- | --- | --- | --- | --- | --- | --- |
|  | APR | LAR | LAR+CAA | Hartmann | Other | W&W | TME | W&W | TME |
| Ayloor[16] | 9 | 1 | NR | NR | NR | NR | NR | NR | NR |
| Dalton[17] | NR | NR | NR | NR | NR | NR | NR | NR | NR |
| Habr[18] | 9 | 6 | 7 | NR | NR | NR | NR | NR | NR |
| Lai[19] | NR | NR | NR | NR | NR | 3.75(4.6) | 1.94(1.1) | 2.31(1.3) | 1.97(0.9) |
| Li[20] | 40 | 30 | 22 | NR | NR | NR | NR | NR | NR |
| Mass[21] | 6 | 14 | NR | NR | NR | NR | NR | NR | NR |
| Smith[22] | NR | NR | NR | NR | NR | NR | NR | NR | NR |
| Wang[23] | 47 | 132 | NR | NR | NR | 2.7  (0.5-36.0) | 3.5  (0.5-186.7) | NR | NR |
| Wang[28] | 32 | NR | NR | NR | NR | NR | NR | NR | NR |

TME: total mesorectal excision; APR: abdominal-perineal resection;; LAR: Low anterior resection; CAA:coloanal anastomosis

NCRT neoadjuvant chemoradiotherapy; NR:no record.
