# Supplementary material for: A meta-analysis of the watch-and-wait strategy versus total mesorectal excision for rectal cancer exhibiting complete clinical response after neoadjuvant chemoradiotherapy
Source: World J Surg Oncol. 2021 Oct 18;19:305. doi: 10.1186/s12957-021-02415-y (PMC8522111; doi:10.1186/s12957-021-02415-y)
Supplement: Supplementary file 9 — Additional file 9. The details of salvage therapy. [file 12957_2021_2415_MOESM9_ESM.doc]

**Supplementary material 9: The details of salvage therapy**

| **Study** | **The details of salvage therapy** |
| --- | --- |
| Ayloor[16] | Seven patients had local recurrence in the W&W group and the mean recurrence interval was 12 months. Five patient were submitted to salvage rectal resection(three APRs, one LAR and one CAA). The mean overall survival of the five patients with salvage rectal resection was 66 months, while The mean overall survival of the two patients refused salvage rectal resection was 21 months. |
| Dalton[17] | Six with CCR did not have surgery and had no evidence of disease within a year of finishing CRT. They have been followed for a mean of 25.5 months (range 12–45 months) and are disease free to date. |
| Habr[18] | Two patients (2.8%) developed endoluminal recurrence after 56 and 64 months of CRT completion. The former was treated by transanal full-thickness excision. Pathologic examination revealed a pT1 and the patient is alive without recurrence, with 72 months of follow-up. The latter was managed by salvage brachytherapy and is alive without recurrence after 132 months of follow-up (over 5 years after brachytherapy). |
| Lai[19] | Local recurrence occurred in two patients, which was treated with transanal wide excision with no associated post-operative complications at 14 and 36 months, and the pathology reports were restricted to ypT1. |
| Li[20] | Two (6.7%) patients developed LR 18 and 26 months after completing NCRT. The first was treated with salvage  dissection (TME) and was alive without LR or distant metastasis (DM) 37 months after the surgery. The second  was managed with local excision and was alive without LR or DM after 62 months of follow up. |
| Mass[21] | Of the 21 patients with the wait-and-see policy, one developed a small endoluminal local recurrence without nodal recurrence after 22 months of follow-up. Endorectal ultrasound showed a T1 tumor. He was offered a low anterior resection but eventually preferred transanal endoscopic microsurgery, which resulted in a complete resection of the recurrence. |
| Smith[22] | One local recurrence, detected 9.4 months after completion of CRT, was treated by transanal endoscopic microsurgery with positive margins followed by APR which showed no evidence of malignancy. This patient developed a subsequent presacral recurrence treated with CyberKnife radiation. He initially had no evidence of disease on PET but developed a pelvic sidewall recurrence four months later. He refused further surgery, and the lesion has been stable on follow-up imaging. He is currently alive with stable disease. |
| Wang[23] | Salvage rectal resection treatment was performed on 7 patients with local recurrence in the W&W group, including 1 case with Dixon operation and 4 cases with APRs operation; |
| Wang[24] | Of the 14 patients with local regrowth, 12 (85.7%) had salvaged R0 resection, of whom fve underwent the Miles procedure. Te 3-year non-regrowth LRFS was 98% (95% CI 95–100%). |

TME: total mesorectal excision; APR: abdominal-perineal resection;; LAR: Low anterior resection; CAA:coloanal anastomosis; LR: local recurrence; DM: distant metastasis; NCRT neoadjuvant chemoradiotherapy; LRFS:local recurrence-free survival: NR:no record.
