# Supplementary material for: A meta-analysis of the watch-and-wait strategy versus total mesorectal excision for rectal cancer exhibiting complete clinical response after neoadjuvant chemoradiotherapy
Source: World J Surg Oncol. 2021 Oct 18;19:305. doi: 10.1186/s12957-021-02415-y (PMC8522111; doi:10.1186/s12957-021-02415-y)
Supplement: Supplementary file 10 — Additional file 10. The details of salvage therapy in W&W group. [file 12957_2021_2415_MOESM10_ESM.doc]

Supplementary material 10 The details of salvage therapy in W&W group

| **Study** | **LR case in W&W group** | **Case with**  **salvage therapy** | **Mean recurrence interval (months)** | **TME** | **APRs** | **LAR** | **Other surgery type** | **Mean overall survival (months)** |
| --- | --- | --- | --- | --- | --- | --- | --- | --- |
| Ayloor[16] | 7(30.13%) | 5(71.43%) | 12 | NR | 3 | 1 | 1 | 66 |
| Dalton[17] | NR | NR | NR | NR | NR | NR | NR | NR |
| Habr[18] | 2(2.81%) | 2(100%) | 60 | NR | NR | NR | 2 | 100 |
| Lai[19] | 2(11.11%) | 2(100%) | 25 | NR | NR | NR | 2 | NR |
| Li[20] | 2(6.66%) | 2(100%) | 22 | 1 | NR | NR | 1 | 49.5 |
| Mass[21] | 1(4.76%) | 1(100%) | 22 | NR | NR | 1 | NR | NR |
| Smith[22] | 1(5.55%) | 1(100%) | 9.4 | NR | NR | NR | 1 | NR |
| Wang[23] | 7(11.86%) | 5(71.43%) | NR | NR | 4 | 1 | NR | NR |
| Wang[24] | 14(14.89%) | 12(85.71%) | NR | NR | 5 | NR | NR | NR |
| Sum | 36(10.81%) | 30(83.33%) | NR | 1 | 12 | 3 | 7 | NR |

LR: local recurrence; TME: total mesorectal excision; APR: abdominal-perineal resection;; LAR: Low anterior resection; CAA: coloanal anastamosis. NR:No record.
