# Supplementary material for: A meta-analysis of the watch-and-wait strategy versus total mesorectal excision for rectal cancer exhibiting complete clinical response after neoadjuvant chemoradiotherapy
Source: World J Surg Oncol. 2021 Oct 18;19:305. doi: 10.1186/s12957-021-02415-y (PMC8522111; doi:10.1186/s12957-021-02415-y)
Supplement: Supplementary file 11 — Additional file 11. The complications of salvage therapy in W&W group and TME group. [file 12957_2021_2415_MOESM11_ESM.doc]

**Supplementary material 11:The complications of salvage therapy in W&W group and TME group**

| **Study** | **W&W group** | **TME group** | | | | | | |
| --- | --- | --- | --- | --- | --- | --- | --- | --- |
|  |  | **hernia** | **Wound**  **infections** | **intra-abdominal**  **abscess** | **fistula** | **hypovolemic shock** | **urinary retention** | **other** |
| Ayloor[16] | NR | NR | NR | NR | NR | NR | NR | NR |
| Dalton[17] | NR | NR | NR | NR | NR | NR | NR | NR |
| Habr[18] | 0 | 0 | 0 | 0 | 0 | 0 | 0 | 0 |
| Lai[19] | 0 | 2 | 5 | 1 | 1 | 1 | 0 | 3 |
| Li[20] | 0 | NR | NR | NR | 62 | NR | NR | NR |
| Mass[21] | 0 | NR | NR | 4 | 3 | NR | 3 | NR |
| Smith[22] | 0 | 5 | 3 | 2 | 6 | NR | NR | 6 |
| Wang[23] | NR | NR | NR | NR | NR | NR | NR | NR |
| Wang[24] | NR | NR | NR | NR | NR | NR | NR | NR |

TME: total mesorectal excision; APR: abdominal-perineal resection;; LAR: Low anterior resection; NR:No record.
