# Supplementary figures and images for: A meta-analysis of the watch-and-wait strategy versus total mesorectal excision for rectal cancer exhibiting complete clinical response after neoadjuvant chemoradiotherapy
Source: World J Surg Oncol. 2021 Oct 18;19:305. doi: 10.1186/s12957-021-02415-y (PMC8522111; doi:10.1186/s12957-021-02415-y)

the funnel plot

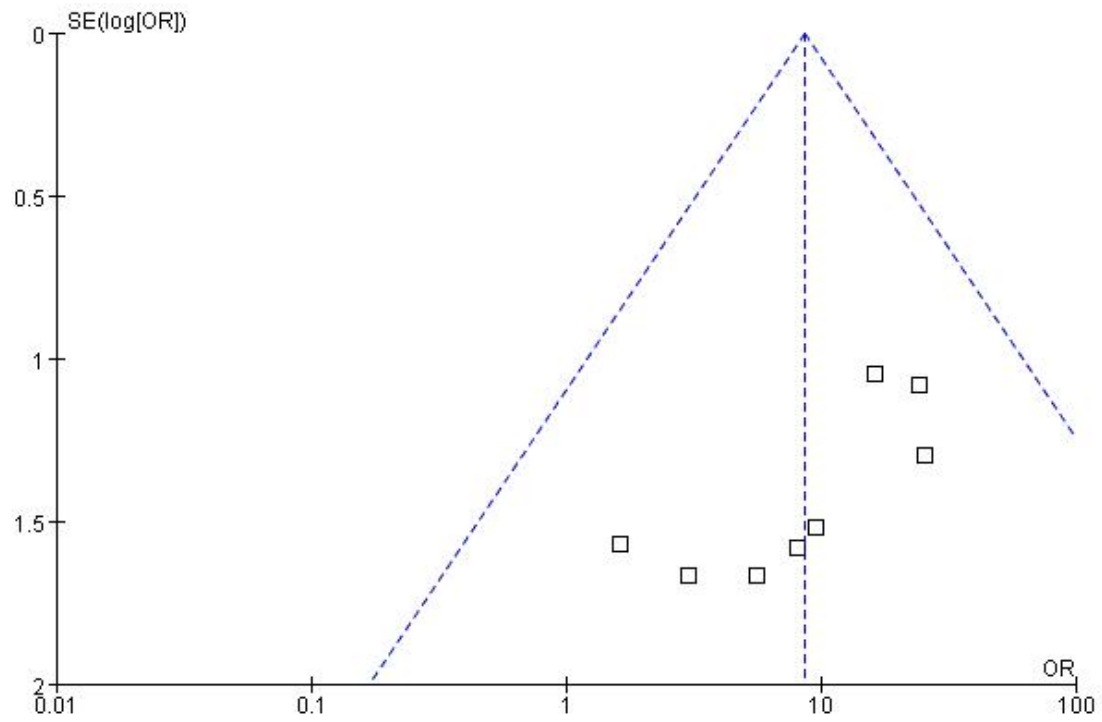

Supplement: Supplementary file 12 — Additional file 12. [file 12957_2021_2415_MOESM12_ESM.pdf]

## 2-DFS in the W&W group was better than that in the TME group

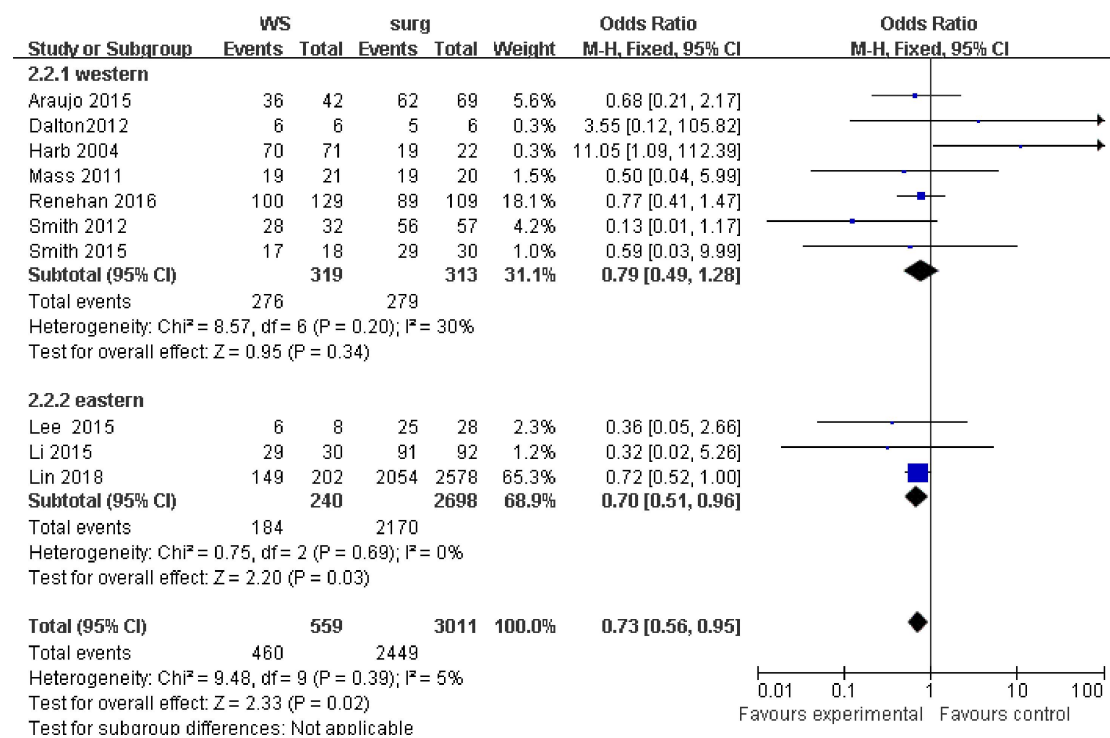

Supplement: Supplementary file 14 — Additional file 14. [file 12957_2021_2415_MOESM14_ESM.pdf]
