# Supplementary material for: A meta-analysis of the watch-and-wait strategy versus total mesorectal excision for rectal cancer exhibiting complete clinical response after neoadjuvant chemoradiotherapy
Source: World J Surg Oncol. 2021 Oct 18;19:305. doi: 10.1186/s12957-021-02415-y (PMC8522111; doi:10.1186/s12957-021-02415-y)
Supplement: Supplementary file 13 — Additional file 13. Comparison between the previous researches. [file 12957_2021_2415_MOESM13_ESM.doc]

**Supplementary material 13:Comparison between the previous researches.**

|  | LR  (OR,95%CI) | DM  (OR,95%CI) | CRD  (OR,95%CI) | 2-DFS  (OR,95%CI) | 2-OS  (OR,95%CI) |
| --- | --- | --- | --- | --- | --- |
| Zhao | 7.32(3.58-14.95) | 1.03(0.59-1.81) | 0.44(0.12-1.60) | 0.77(0.46-1.28) | 0.75(0.40-1.41) |
| Dossa | 15.7(11.8-20.1) | NR | 0.58(0.06-5.84) | 0.56(0.02-1.60) | 3.91(0.57-26.72) |
| Li | 6.97(2.44-19.93) | 0.93(0.44-1.96) | NR | 0.97(0.92-1.03) | 1.02(0.98-1.06) |
| Dattani | 5.37(2.56-11.27) | NR | 0.82(0.46-1.35) | 1.69(1.08-2.64) | NR |
| Our study | 7.44(3.01-18.38) | 1.11(0.66-1.86) | 0.41(0.13-1.34) | 3.13(0.83-11.76) | 1.74(0.48-6.32) |

|  | 3-DFS  (OR,95%CI) | 3-OS  (OR,95%CI) | 5-DFS  (OR,95%CI) | 5-OS  (OR,95%CI) |
| --- | --- | --- | --- | --- |
| Zhao | NR | NR | 0.47(0.04-5.90) | 1.94(0.19-19.62) |
| Dossa | NR | NR | NR | NR |
| Li | 0.95(0.85-1.06) | 1.01(0.97-1.06) | 0.96(0.85-1.08) | 1.01(0.92-1.11) |
| Dattani | NR | NR | NR | NR |
| Our study | 3.17(0.78-12.86) | 1.23(0.20-7.65) | 1.79(0.27-11.80) | 1.03(0.26-4.08) |

LR: local recurrence; DM: distant metastasis; CRD: cancer-related death; DFS: disease-free survival; OS: overall survival (OS); CI: confidence interval; OR: Odds ratios;
