# Supplementary material for: A meta-analysis of the watch-and-wait strategy versus total mesorectal excision for rectal cancer exhibiting complete clinical response after neoadjuvant chemoradiotherapy
Source: World J Surg Oncol. 2021 Oct 18;19:305. doi: 10.1186/s12957-021-02415-y (PMC8522111; doi:10.1186/s12957-021-02415-y)
Supplement: Supplementary file 15 — Additional file 15. Editing certificate. A meta-analysis of the watch-and-wait strategy versus total mesorectal excision for rectal cancer exhibiting complete clinical response after. [file 12957_2021_2415_MOESM15_ESM.pdf]

Editing Certificate

This document certifies that the manuscript

A meta-analysis of the watch-and-wait strategy versus total mesorectal excision for rectal cancer exhibiting complete clinical response after...

was edited for proper English language, grammar, punctuation, spelling, and overall style by one or more of the highly qualified native English speaking editors at AJE.

This certificate was issued on April 21, 2021 and may be verified on the AJE website using the verification code 9A1B-402D-72FD-53CA-D156.

Neither the research content nor the authors' intentions were altered in any way during the editing process. Documents receiving this certification should be English-ready for publication; however, the author has the ability to accept or reject our suggestions and changes. To verify the final AJE edited version, please visit our verification page at aje.com/certificate. If you have any questions or concerns about this edited document, please contact AJE at support@aje.com.

AJE provides a range of editing, translation, and manuscript services for researchers and publishers around the world. For more information about our company, services, and partner discounts, please visit aje.com.
