# Supplementary material for: A meta-analysis of the watch-and-wait strategy versus total mesorectal excision for rectal cancer exhibiting complete clinical response after neoadjuvant chemoradiotherapy
Source: World J Surg Oncol. 2021 Oct 18;19:305. doi: 10.1186/s12957-021-02415-y (PMC8522111; doi:10.1186/s12957-021-02415-y)
Supplement: Supplementary file 17 — Additional file 17: Fig 1. Flowchart of the included studies. Fig 2. Outcomes of W&W group versus TME group. a. local recurrence; b: distant metastasis; c: cancer related death. Fig 3. Outcomes of W&W group versus TME group. a. 2-year DFS; b: 2-year OS; c: 3-year DFS; d: 3-year OS; e: 5-year DFS; f: 5-year OS. [file 12957_2021_2415_MOESM17_ESM.doc]

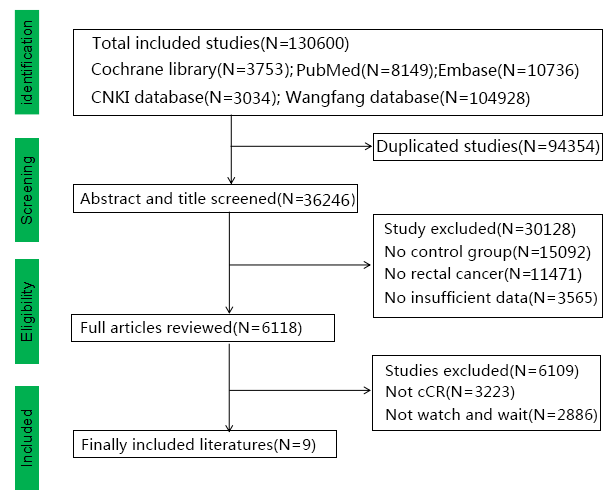


**Fig 1 Flowchart of the included studies**

**
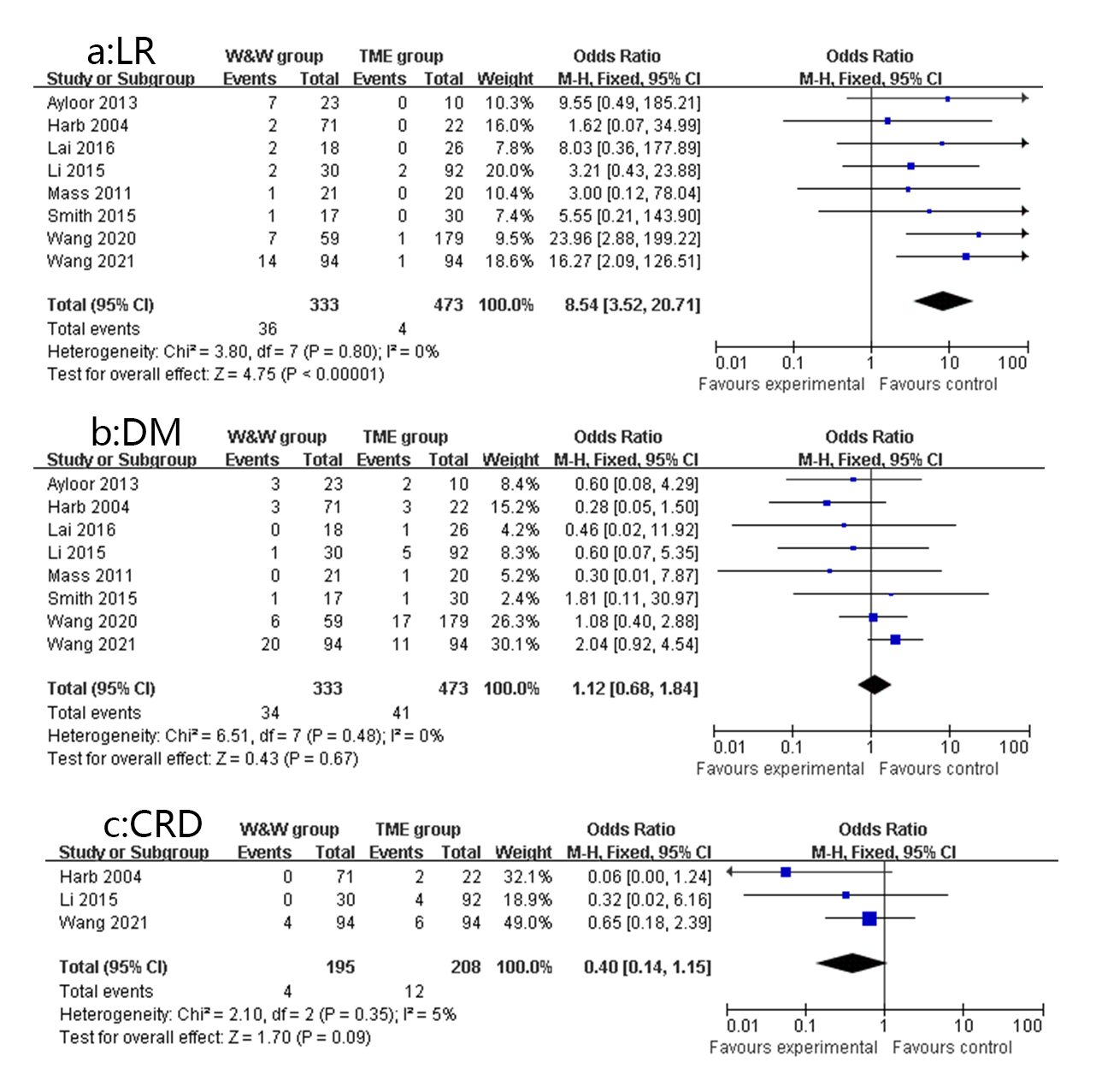
**

**Fig 2 Outcomes of W&W group versus TME group. a. local recurrence; b: distant metastasis; c: cancer related death**


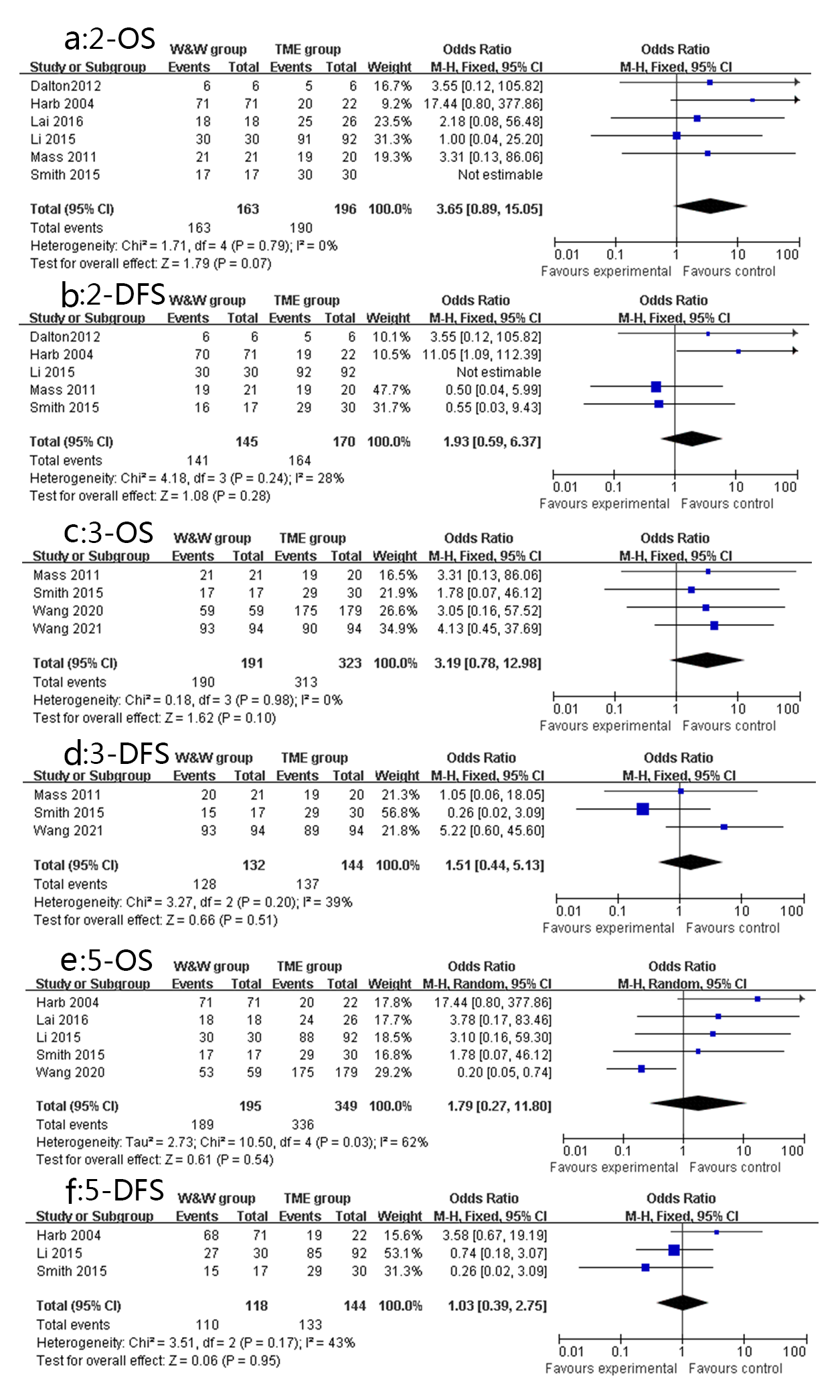


**Fig 3 Outcomes of W&W group versus TME group. a. 2-year DFS; b: 2-year OS; c: 3-year DFS; d: 3-year OS; e: 5-year DFS; f: 5-year OS**
