# Supplementary material for: A meta-analysis of the watch-and-wait strategy versus total mesorectal excision for rectal cancer exhibiting complete clinical response after neoadjuvant chemoradiotherapy
Source: World J Surg Oncol. 2021 Oct 18;19:305. doi: 10.1186/s12957-021-02415-y (PMC8522111; doi:10.1186/s12957-021-02415-y)
Supplement: Supplementary file 18 — Additional file 18. Statistical method. [file 12957_2021_2415_MOESM18_ESM.doc]

**Supplementary material 18: statistical method**

| Study | **statistical method** |
| --- | --- |
| Ayloor[16] | NR |
| Dalton[17] | NR |
| Habr[18] | Statistical analysis was performed using χ2, Student *t* test, and Kaplan-Meier curves for survival analysis. Signifcance was defned as a two-sided *P*<0.05. |
| Lai[19] | Univariate associations bettween categorical variables were assessed using Fisher’s exact test. The 5-year overall survival and disease-free survival rates were calculated with the Kaplan–Meier method. Signifcance was defned as a two-sided *P*<0.05. |
| Li[20] | Information on baseline characteristics was collected and compared using χ2 and *t* tests. Signifcance was defned as a two-sided *P*<0.05. |
| Mass[21] | Mean scores were compared between the wait-and-see patients and pCR control group with the independent samples *t* test to compare continuous variables and χ2 test to compare proportions. Signifcance was defned as a two-sided *P*<0.05. |
| Smith[22] | Where applicable, univariate associations between categorical variables were assessed using Fisher’s exact test given the cell counts of less than five. Two-tailed, unpaired Student’s *t* tests were used to compare the means of continuous variables. Signifcance was defned as a two-sided *P*<0.05. |
| Wang[23] | The measurement data that does not conform to the normal distribution are represented by M (range), using the Wilcoxon rank sum test; the count data is represented by example (%), the comparison between the two groups uses the χ2 test, and the comparison between the two groups of grade data uses the rank sum test. Signifcance was defned as a two-sided *P*<0.05. |
| Wang[24] | Categorical variables were described in terms of frequency, and compared using the χ2 test. Non-normally distributed continuous variables were described as medians, and compared using the Mann–Whitney U test.Statistical analyses were performed using the Statistical Package for the Social Sciences Program (SPSS, Chicago, IL, version 24.0). Signifcance was defned as a two-sided *P*<0.05. |

TME: total mesorectal excision; APR: abdominal-perineal resection;; LAR: Low anterior resection; CAA:coloanal anastomosis

NCRT neoadjuvant chemoradiotherapy; NR:no record.
