# Supplementary material for: A meta-analysis of the watch-and-wait strategy versus total mesorectal excision for rectal cancer exhibiting complete clinical response after neoadjuvant chemoradiotherapy
Source: World J Surg Oncol. 2021 Oct 18;19:305. doi: 10.1186/s12957-021-02415-y (PMC8522111; doi:10.1186/s12957-021-02415-y)
Supplement: Supplementary file 19 — Additional file 19. the details of adjuvant chemotherapy of studies. [file 12957_2021_2415_MOESM19_ESM.doc]

**Supplementary material 19: the details of adjuvant chemotherapy of studies**

| Study | adjuvant chemotherapy |
| --- | --- |
| Ayloor[16] | NR |
| Dalton[17] | NR |
| Habr[18] | NR |
| Lai[19] | NR |
| Li[20] | NR |
| Mass[21] | Oxaliplatin 130 mg/m2 on day 1 and capecitabine 1,000 mg/m^2^ twice daily from day 1 to 14. |
| Smith[22] | NR |
| Wang[23] | Chemotherapy regimens include fluorouracil or capecitabine single-drug regimen, or combined chemotherapy regimens based on fluorouracil or capecitabine, etc. |
| Wang[24] | Oxaliplatin in combination with capecitabine or 5-furouracil. |

TME: total mesorectal excision; APR: abdominal-perineal resection;; LAR: Low anterior resection; CAA:coloanal anastomosis

NCRT neoadjuvant chemoradiotherapy; NR:no record.
