# Supplementary material for: A meta-analysis of the watch-and-wait strategy versus total mesorectal excision for rectal cancer exhibiting complete clinical response after neoadjuvant chemoradiotherapy
Source: World J Surg Oncol. 2021 Oct 18;19:305. doi: 10.1186/s12957-021-02415-y (PMC8522111; doi:10.1186/s12957-021-02415-y)
Supplement: Supplementary file 20 — Additional file 20. Search terms and database. [file 12957_2021_2415_MOESM20_ESM.doc]

**We have added new search tools (Ovid database) and tried search terms, but the search results did not contained the nine included articles. We tried other search terms and search tools, but did not achieve the expected results, so we still stick to the original search strategy.**

**We first tried the first set of search terms**

**The search string was “watch-and-wait” and “total mesorectal excision” and “neoadjuvant chemoradiotherapy” and “rectal cancer”.**

**We did not find several included articles.**

**Such as:**

**16. Ayloor Seshadri R, Kondaveeti SS, Jayanand SB, et al. Complete clinical response to neoadjuvant chemoradiation in rectal cancers: can surgery be avoided? Hepatogastroenterology. 2013;60:410-414.**

**17. Dalton RS, Velineni R, Osborne ME, et al. A single-centre experience of chemoradiotherapy for rectal cancer: is there potential for nonoperative management? Colorectal Dis. 2012;14:567-571.**

**the more details were as follows.**

**OVID database**


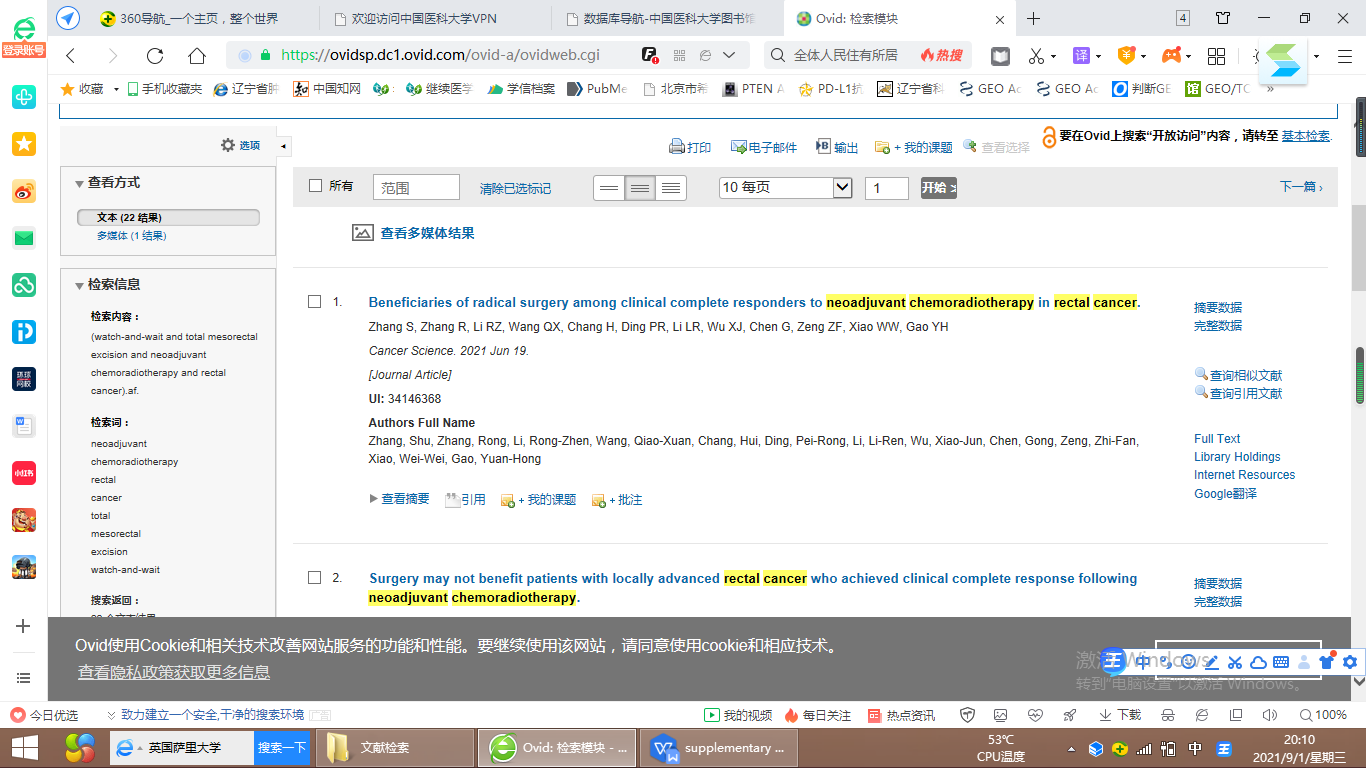


**Embase database**


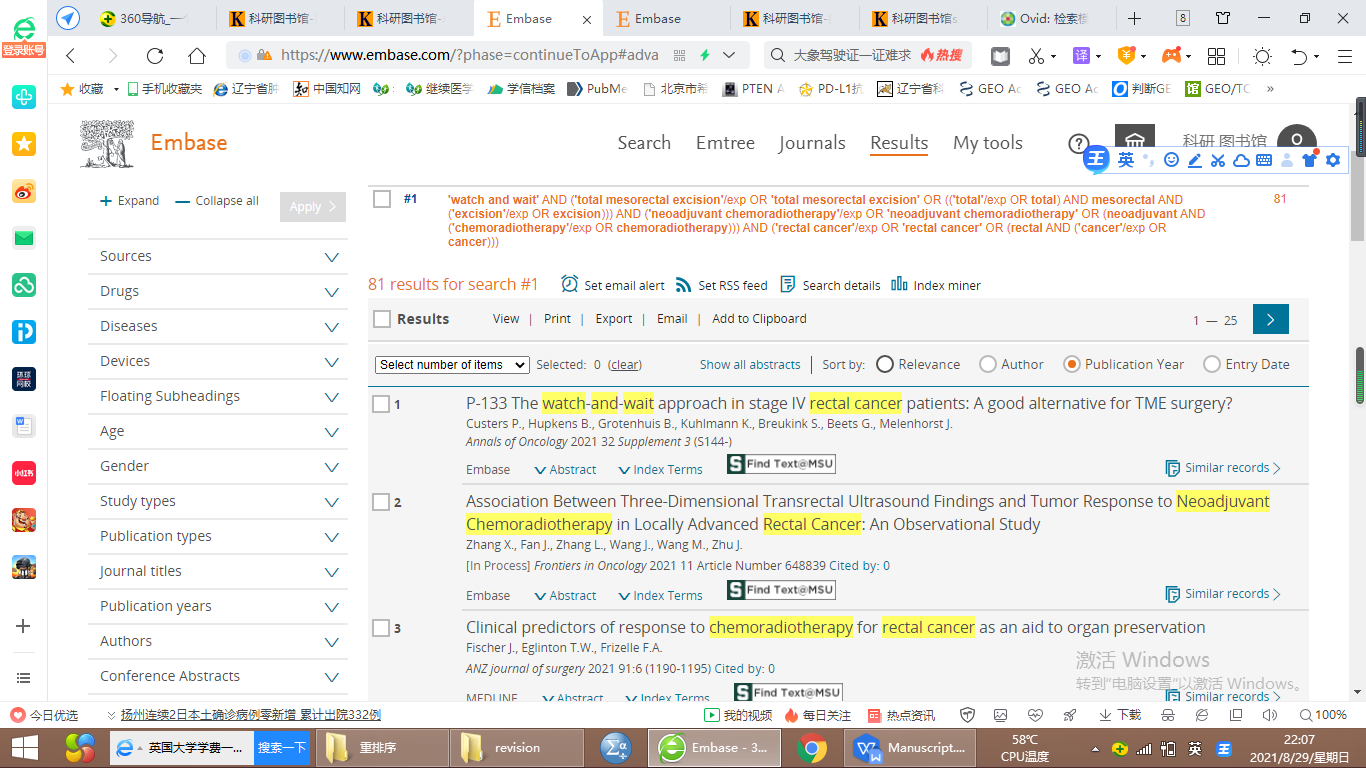


**PubMed database**


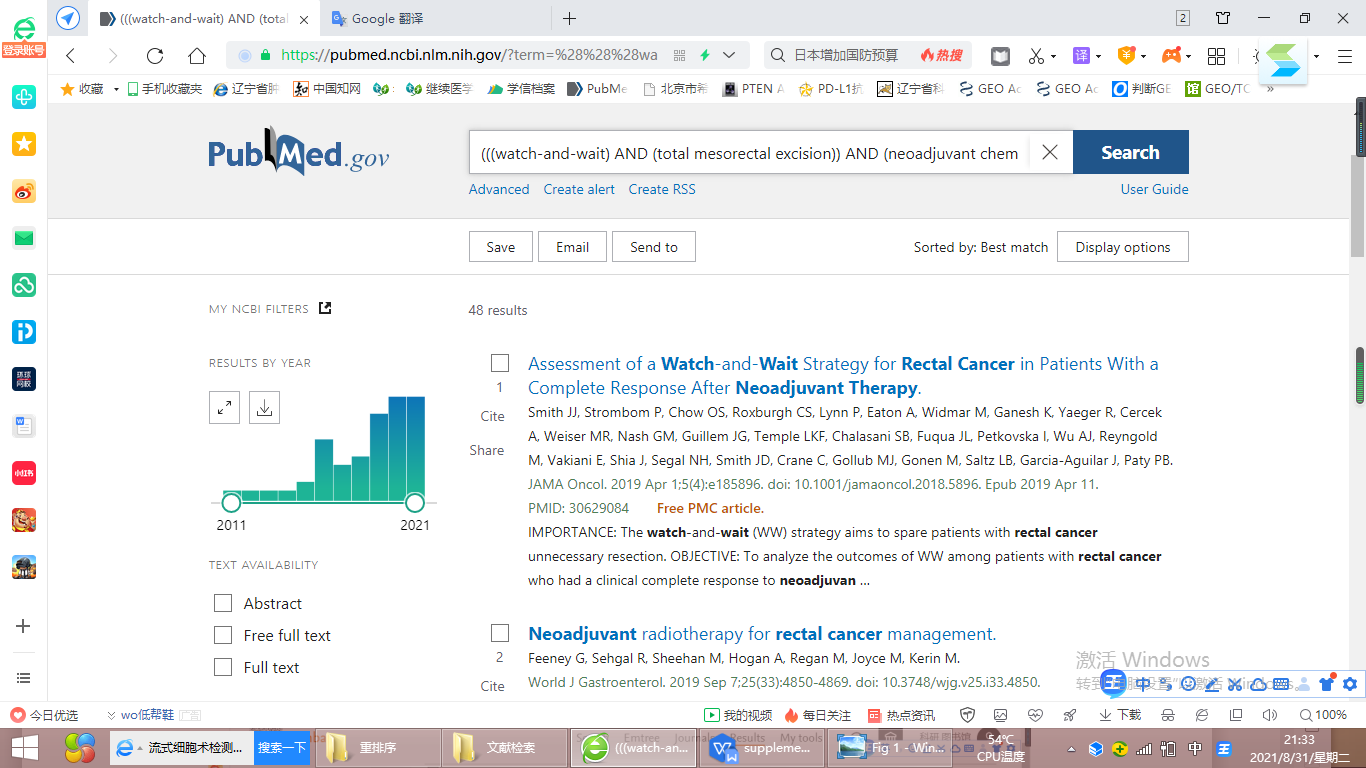


**Cochrane Library**


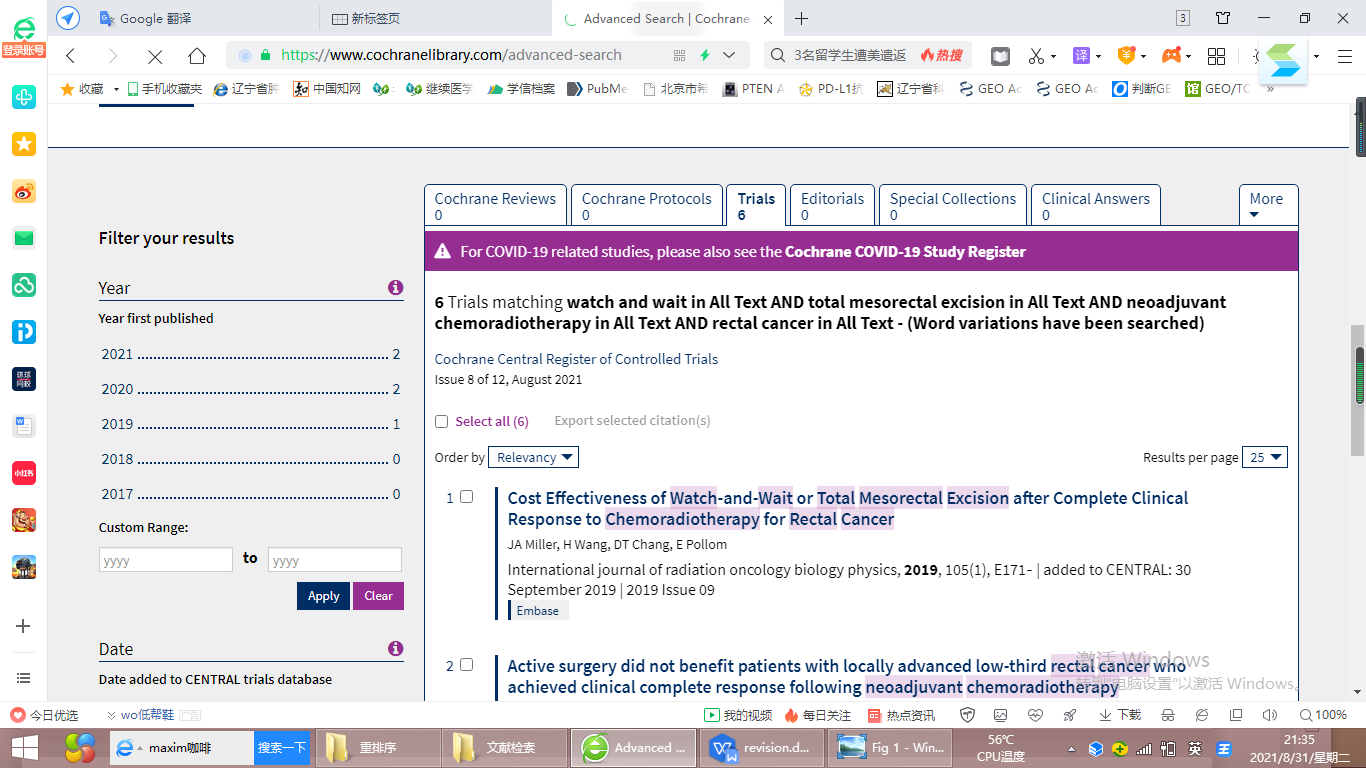


**CNKI database**


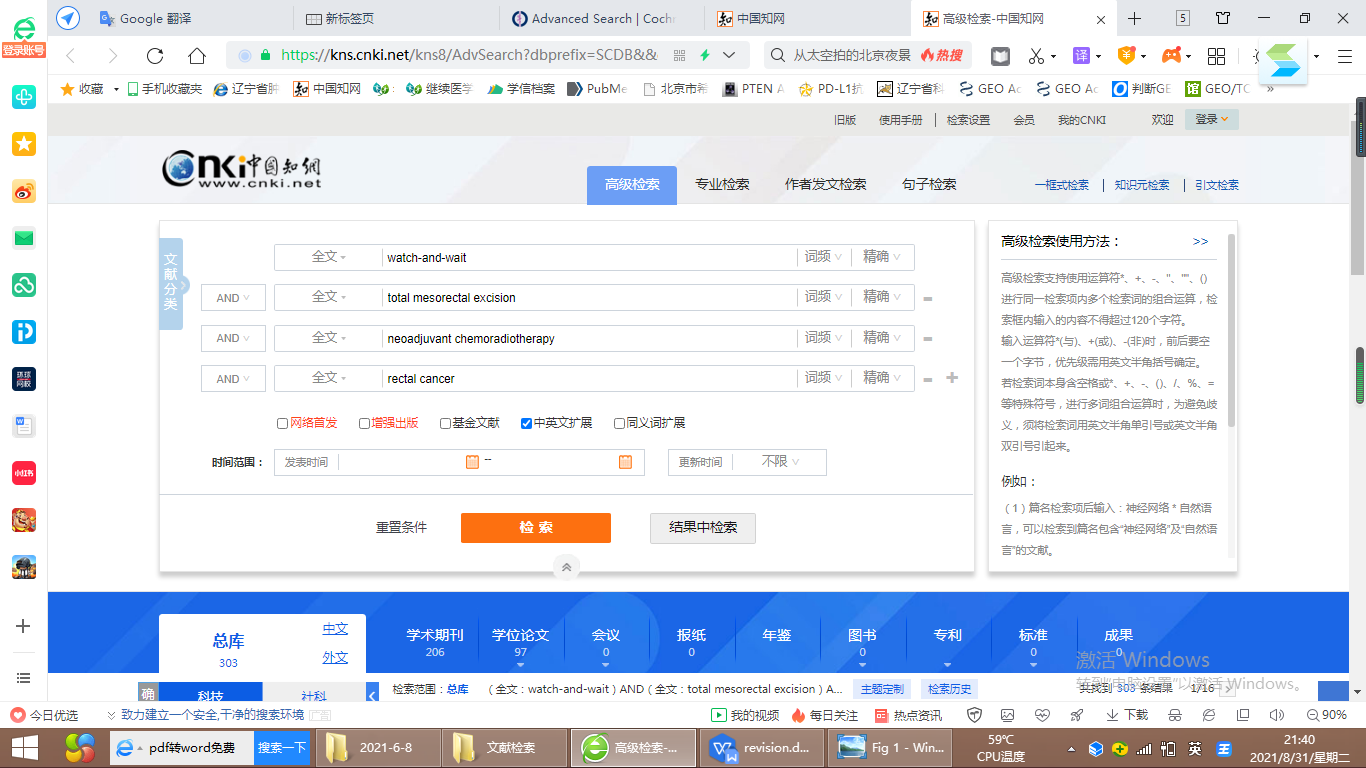


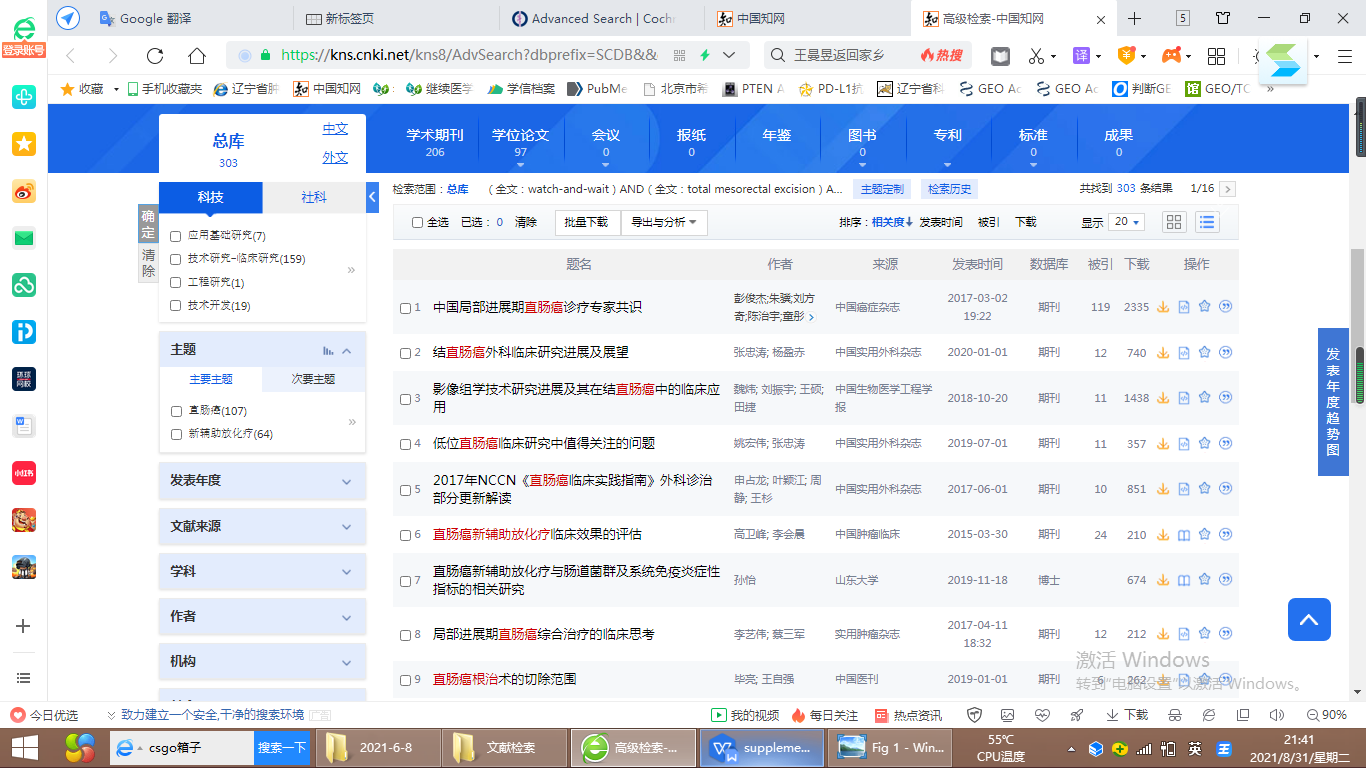


**Wangfang database**


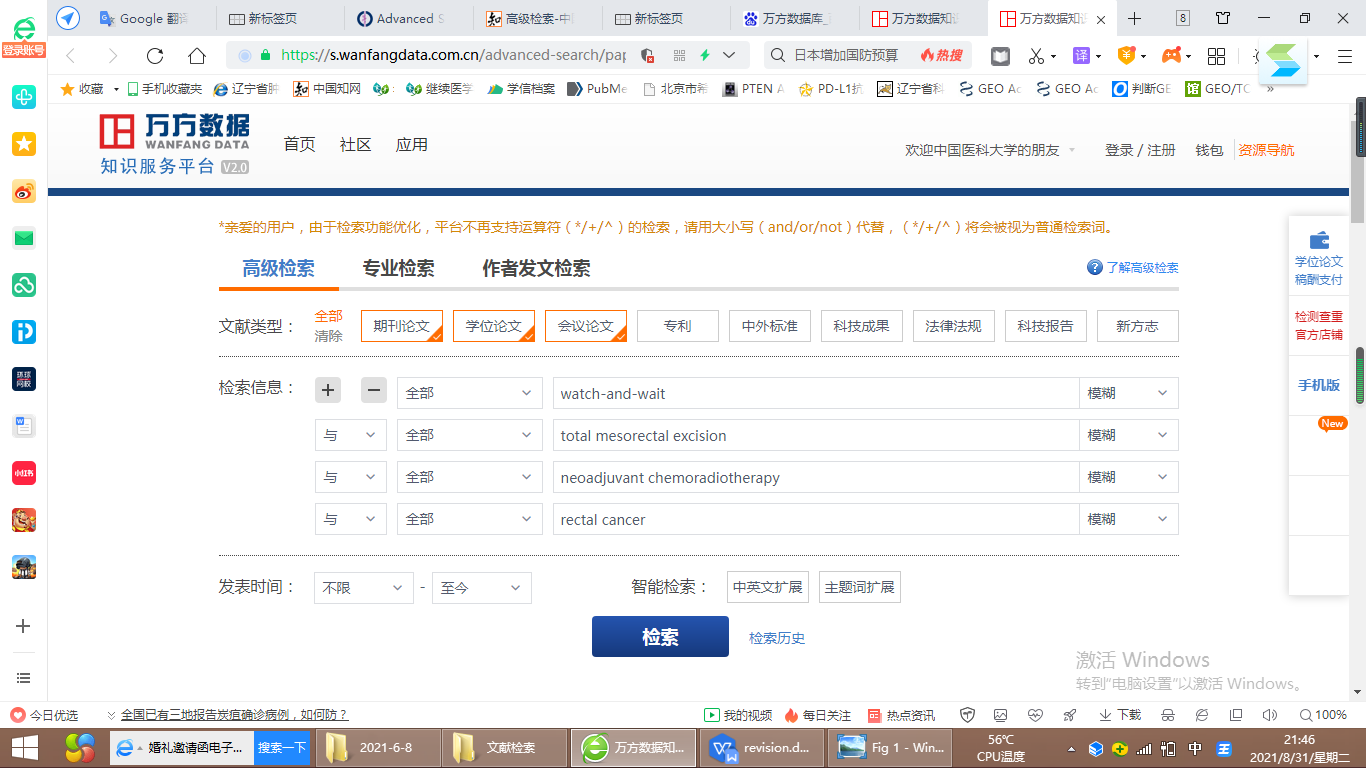


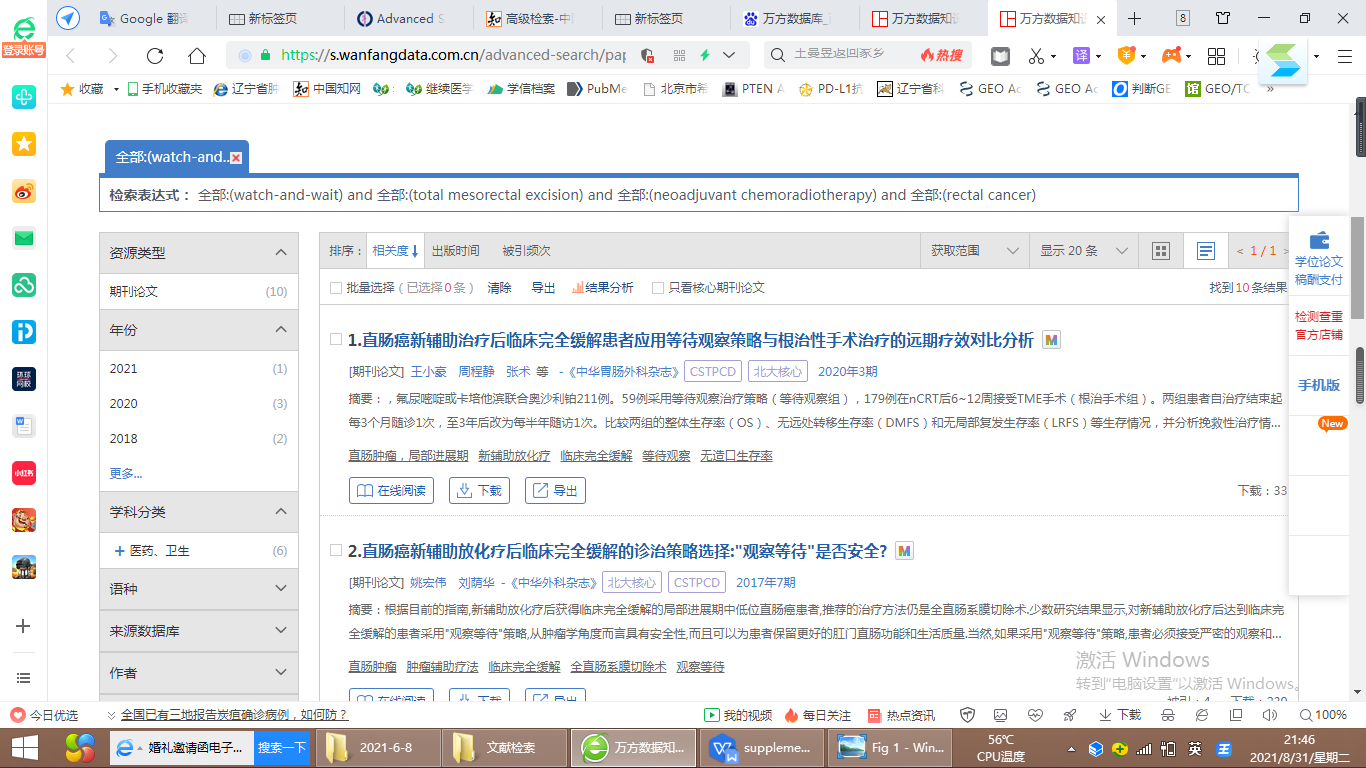


**We first tried the second set of search terms**

**The search string was “watch-and-wait” and “nonoperative management” and “neoadjuvant chemoradiotherapy” and “rectal cancer”.**

**We did not find several included articles.**

**Such as:**

**16. Ayloor Seshadri R, Kondaveeti SS, Jayanand SB, et al. Complete clinical response to neoadjuvant chemoradiation in rectal cancers: can surgery be avoided? Hepatogastroenterology. 2013;60:410-414.**

**17. Dalton RS, Velineni R, Osborne ME, et al. A single-centre experience of chemoradiotherapy for rectal cancer: is there potential for nonoperative management? Colorectal Dis. 2012;14:567-571.**

**21. Maas M, Beets-Tan RG, Lambregts DM, et al. Wait-and-see policy for clinical complete responders after chemoradiation for rectal cancer. J Clin Oncol. 2011;29:4633-4640.**

**the more details were as follows.**

**OVID database**


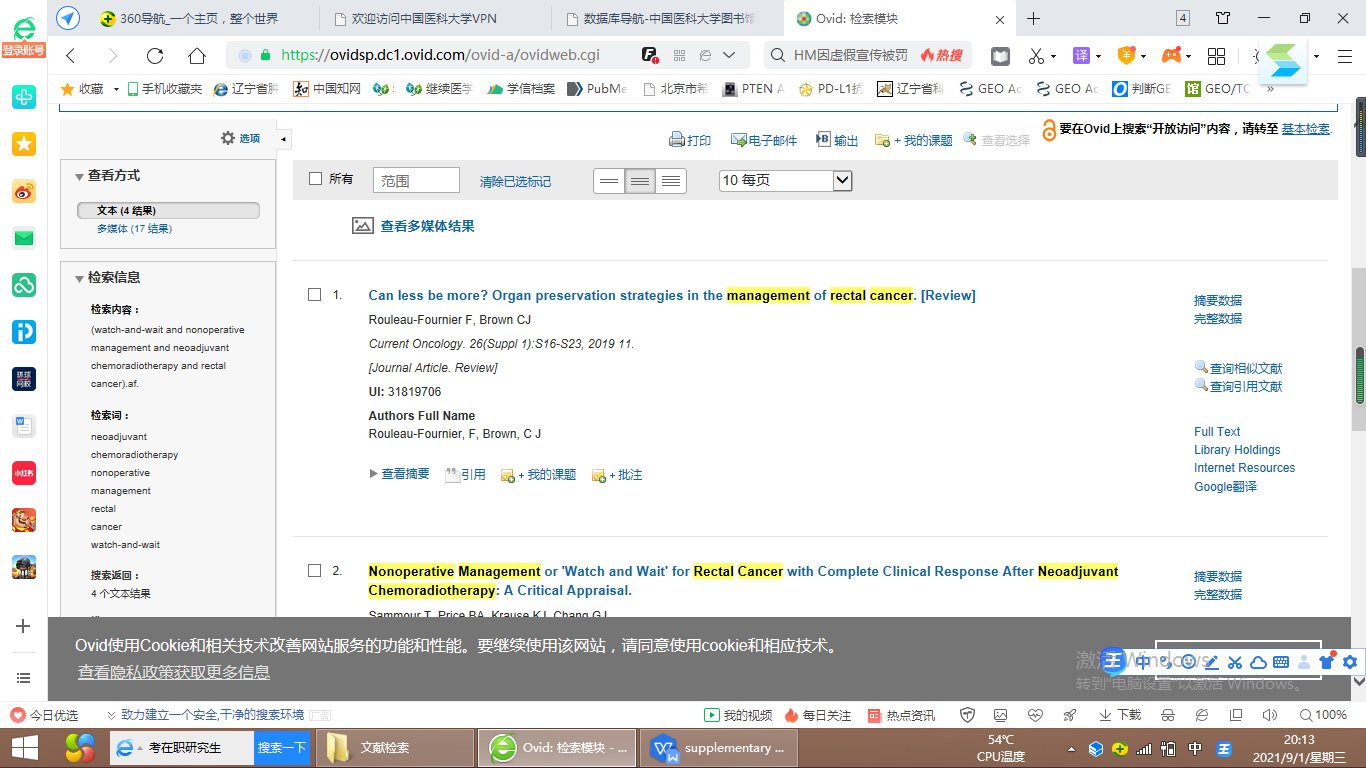


**Embase database**


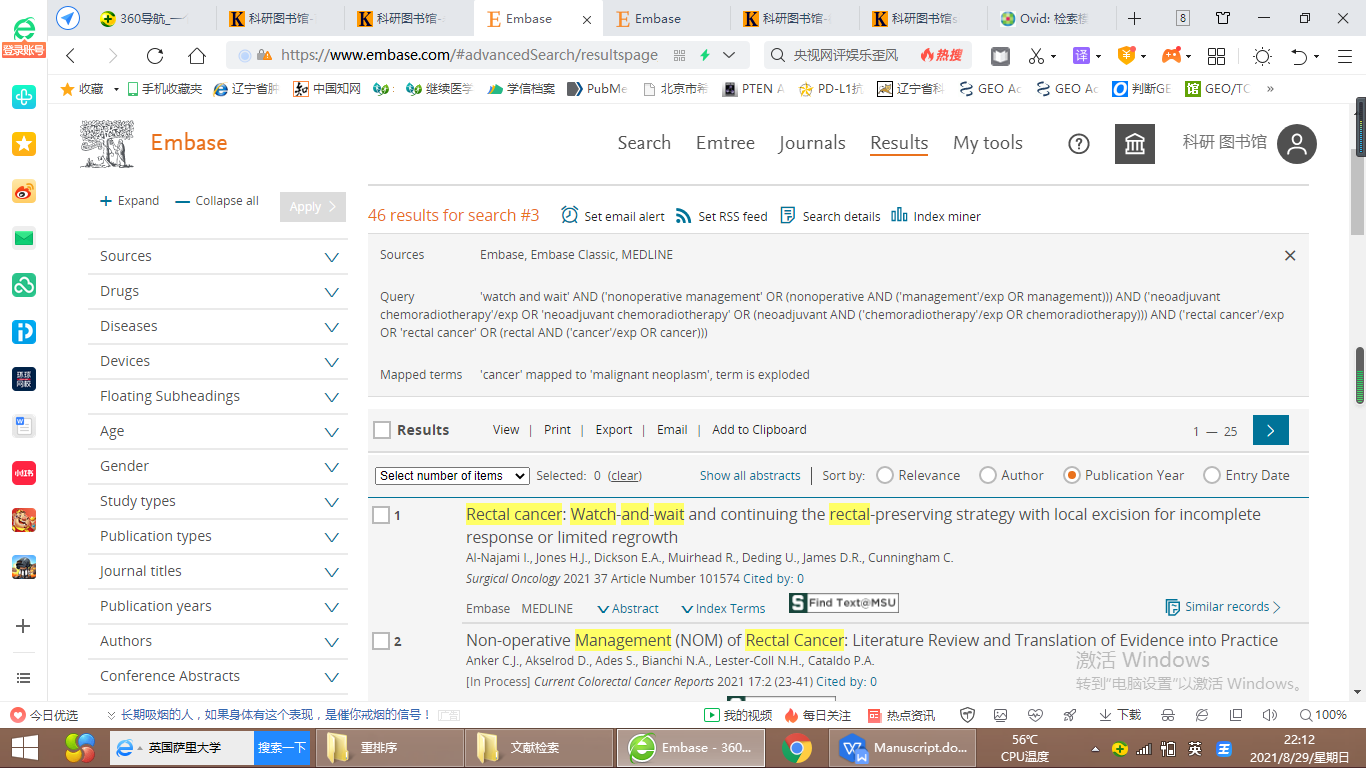


**PubMed database**


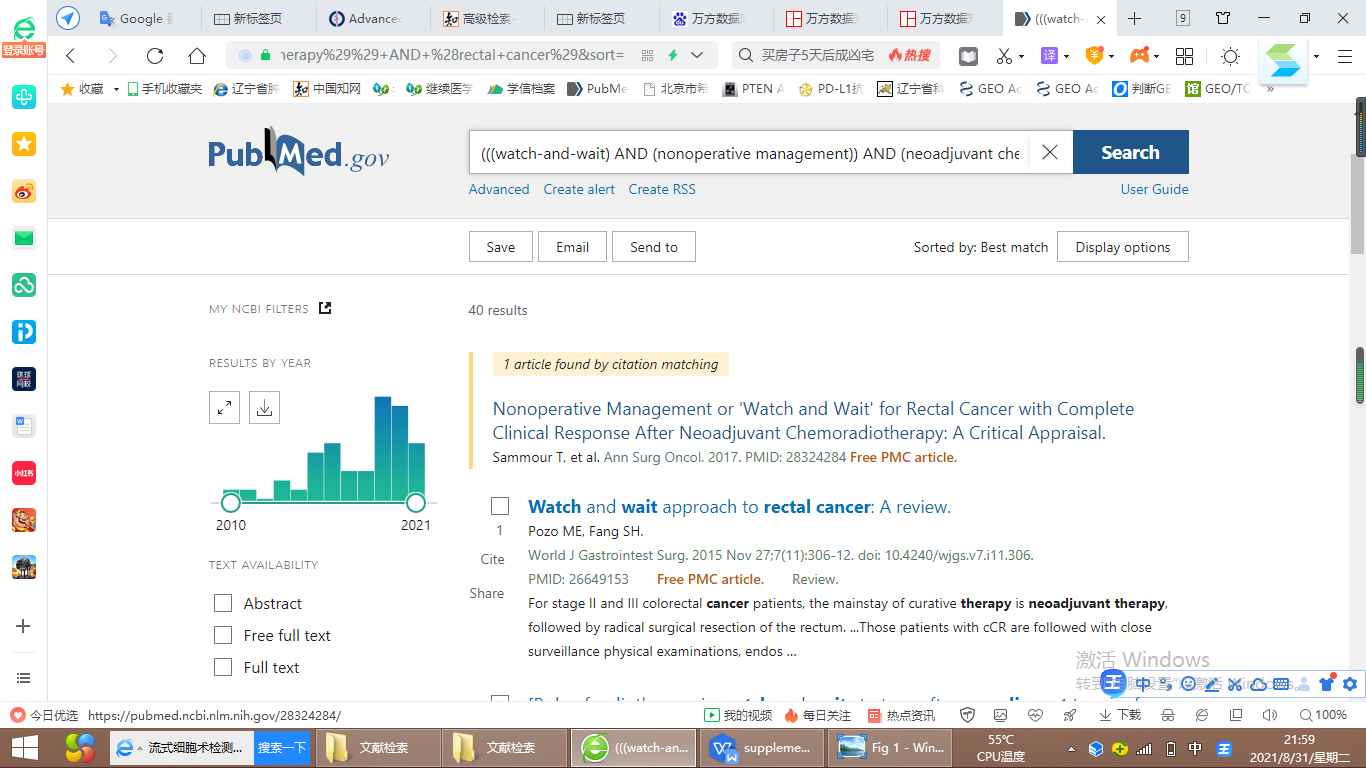


**Cochrane Library**


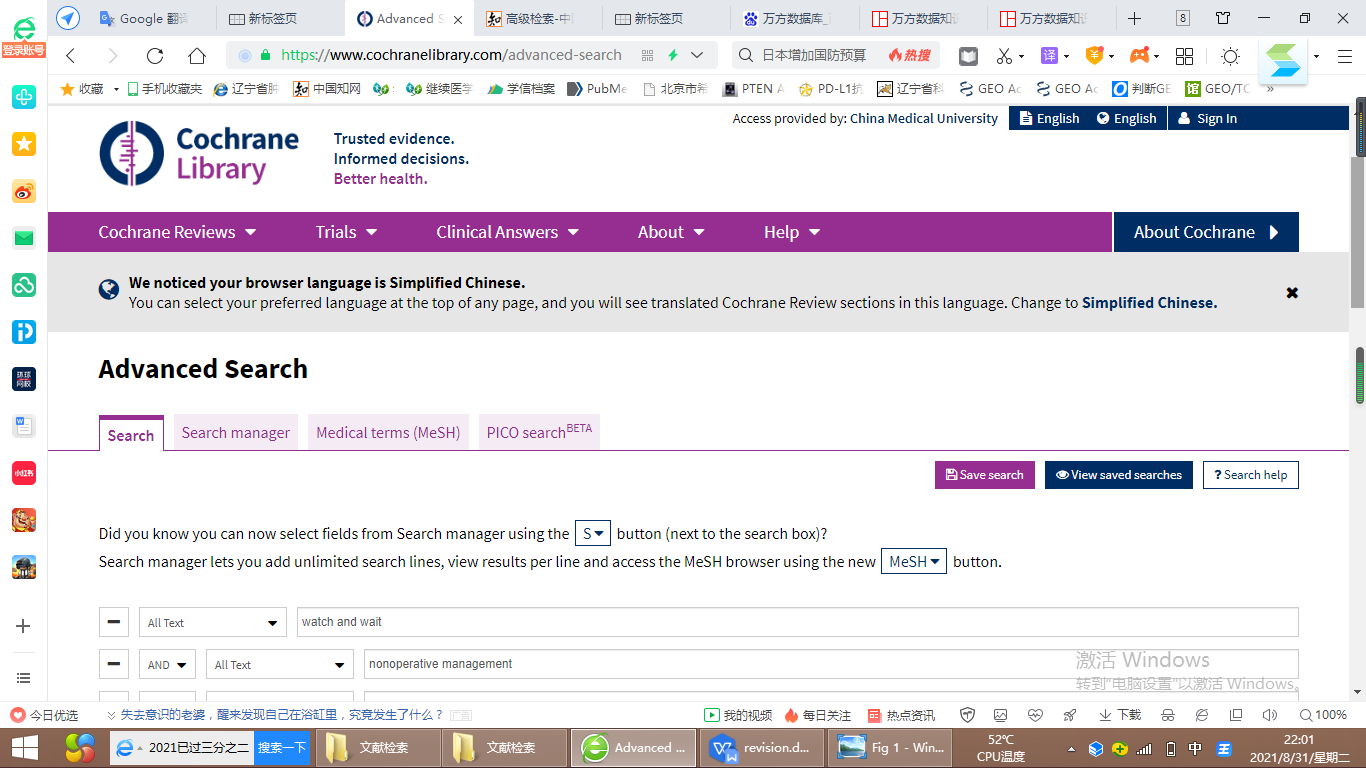

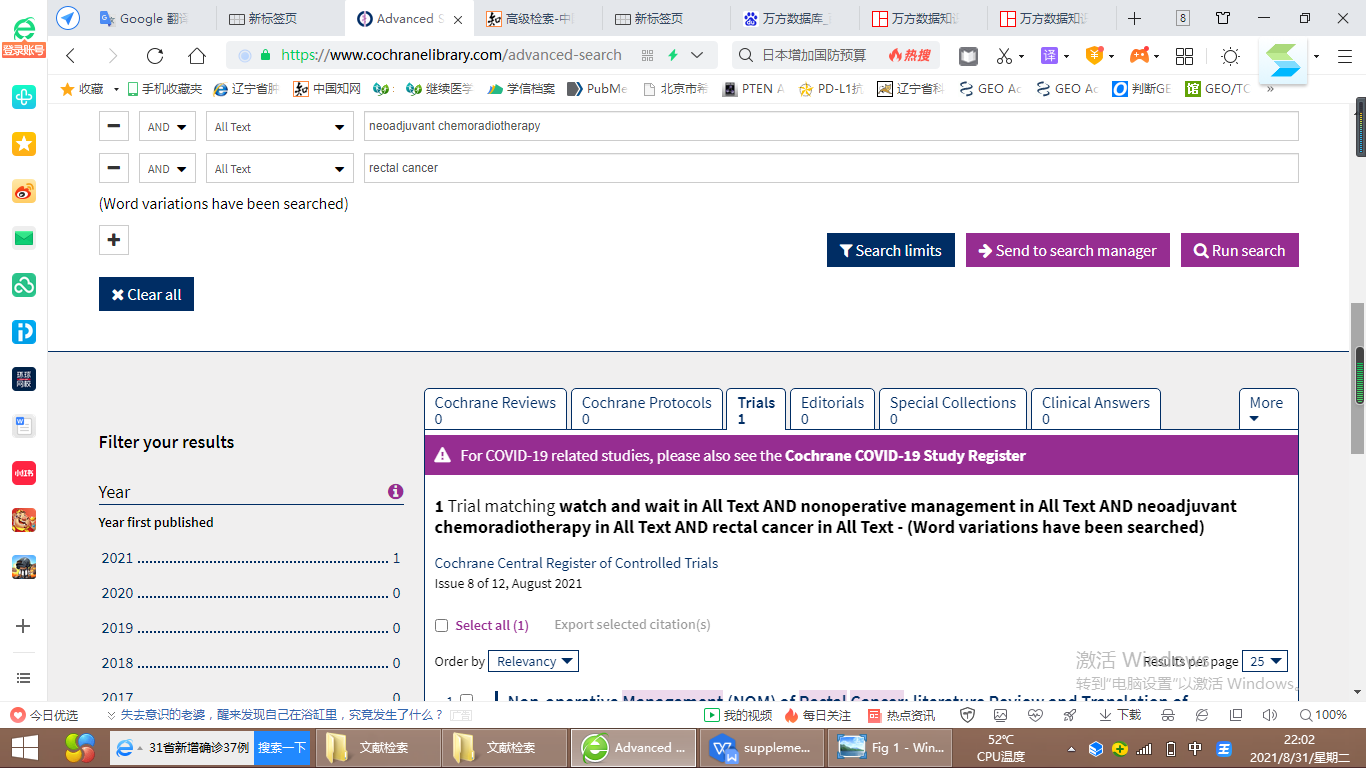


**CNKI database**


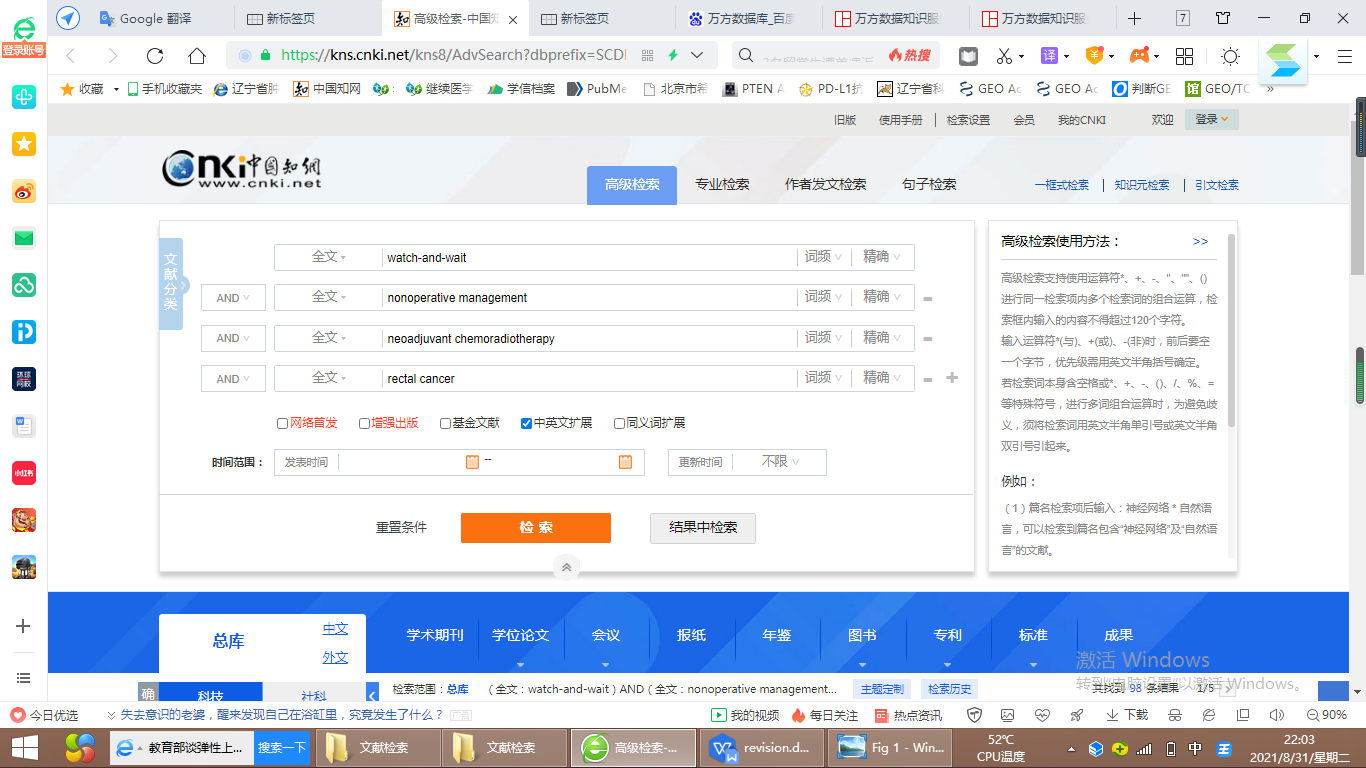


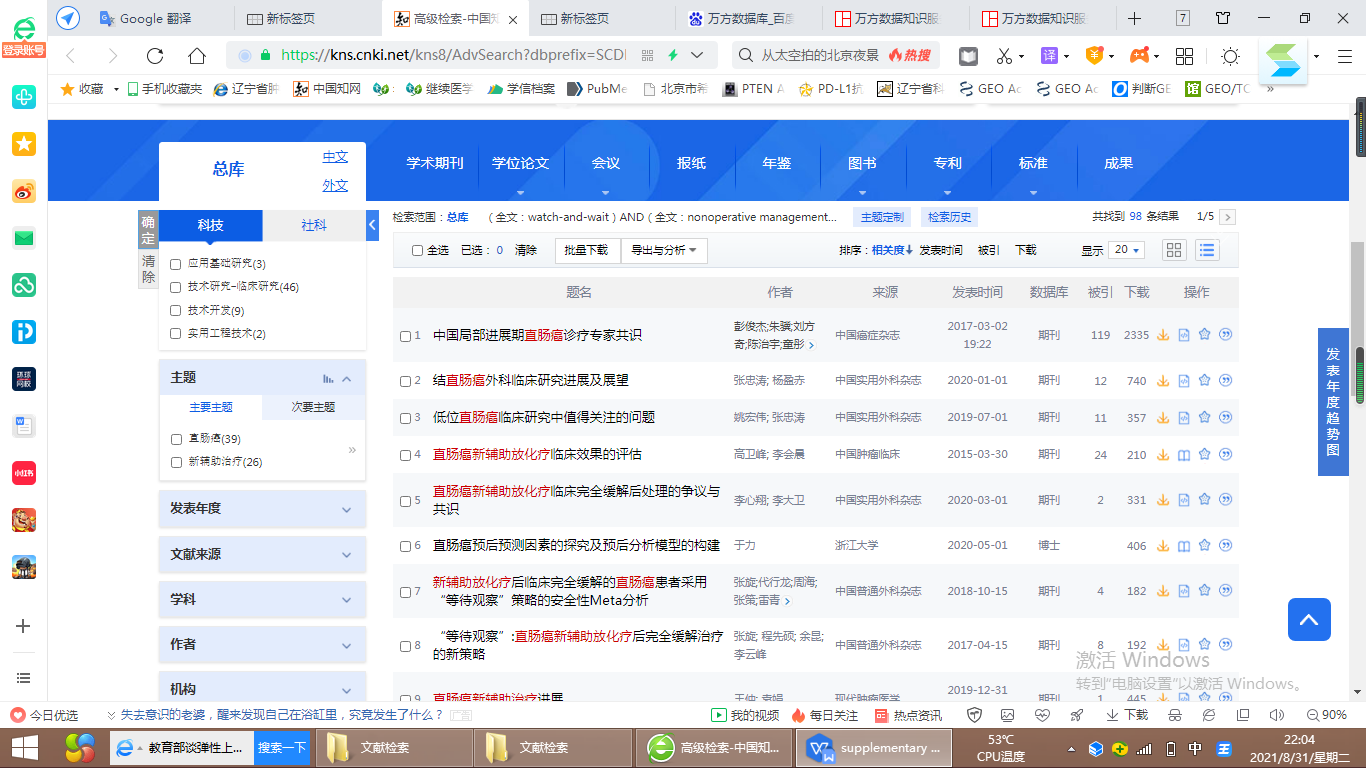


**Wangfang database**


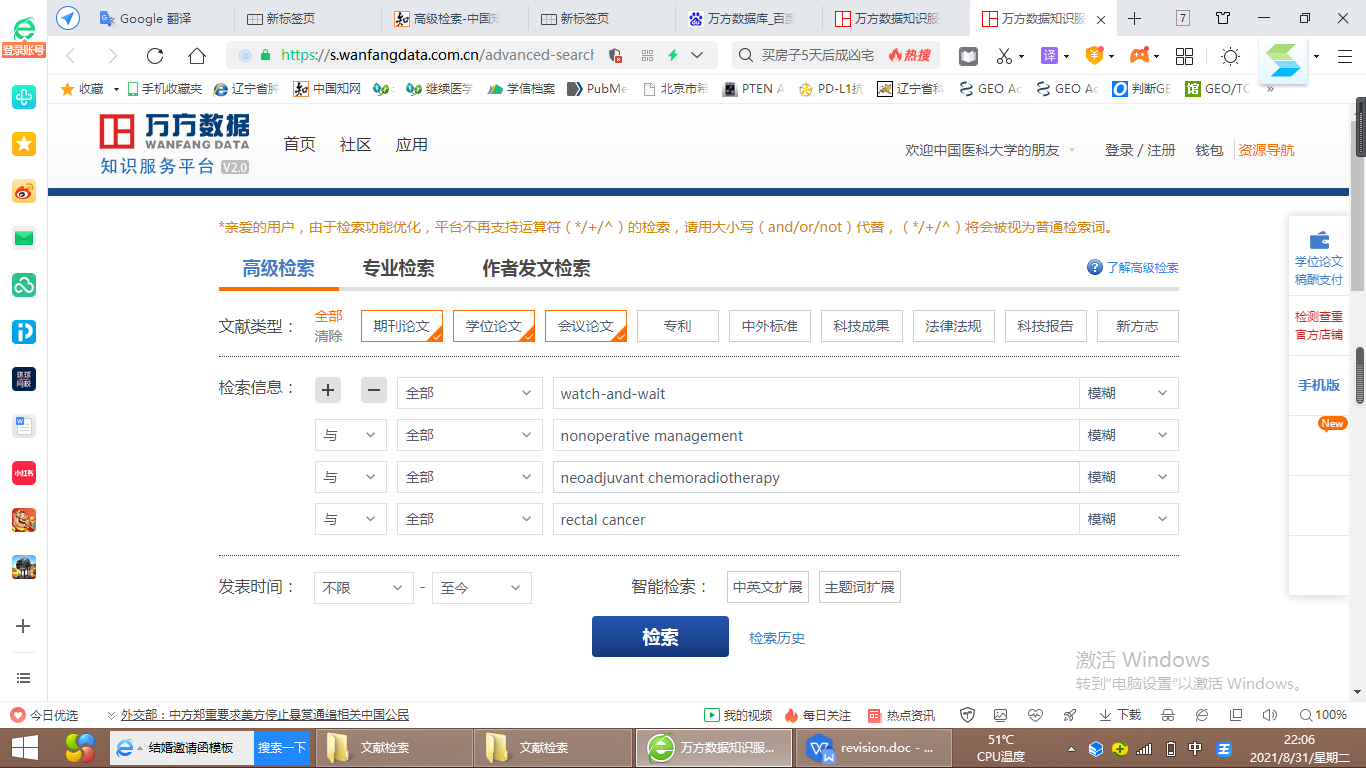


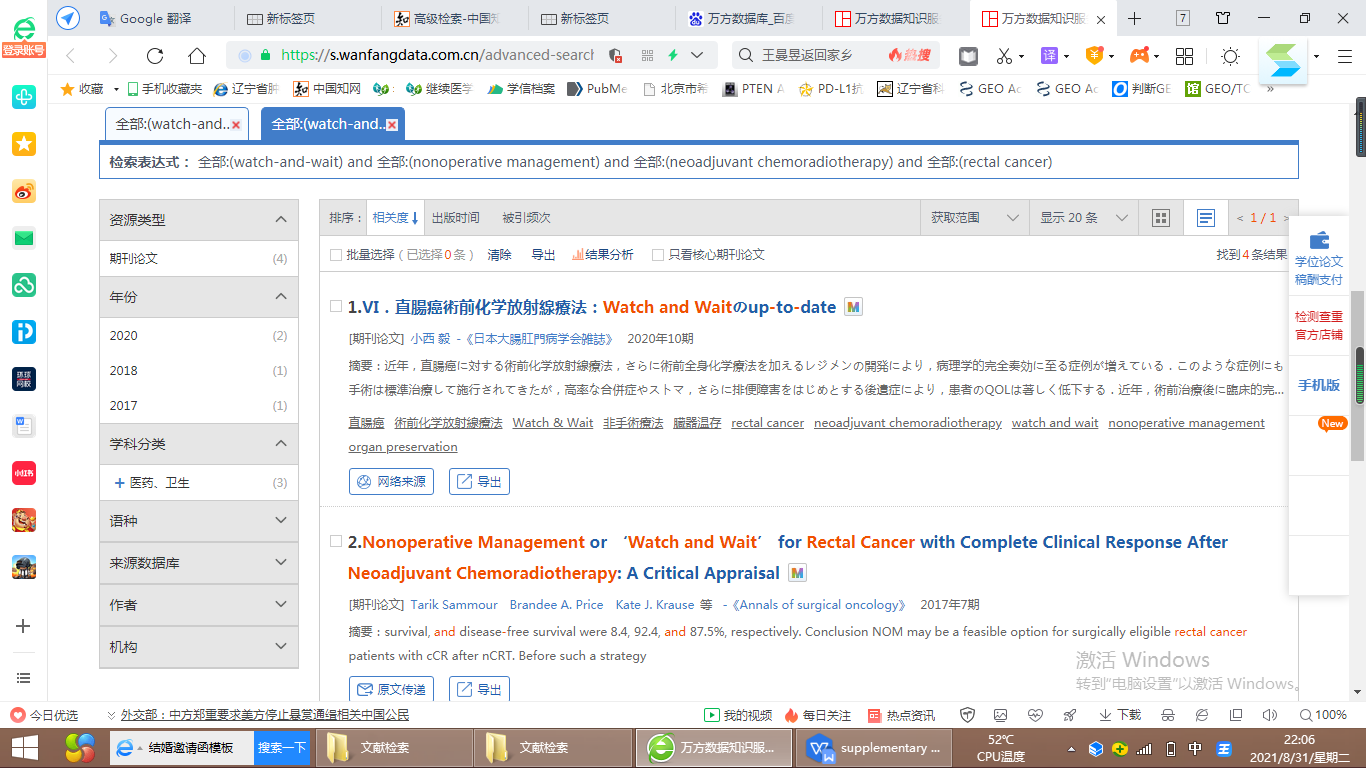


**We tried other search terms and search tools, but did not achieve the expected results, so we still stick to the original search strategy.**
